# Abnormal morphology biases hematocrit distribution in tumor vasculature and contributes to heterogeneity in tissue oxygenation

**DOI:** 10.1073/pnas.2007770117

**Published:** 2020-10-27

**Authors:** Miguel O. Bernabeu, Jakub Köry, James A. Grogan, Bostjan Markelc, Albert Beardo, Mayeul d’Avezac, Romain Enjalbert, Jakob Kaeppler, Nicholas Daly, James Hetherington, Timm Krüger, Philip K. Maini, Joe M. Pitt-Francis, Ruth J. Muschel, Tomás Alarcón, Helen M. Byrne

**Affiliations:** ^a^Centre for Medical Informatics, Usher Institute, The University of Edinburgh, Edinburgh EH16 4UX, United Kingdom;; ^b^Mathematical Institute, University of Oxford, Oxford OX2 6GG, United Kingdom;; ^c^Department of Oncology, University of Oxford, Oxford OX3 7DQ, United Kingdom;; ^d^Department of Experimental Oncology, Institute of Oncology Ljubljana, 1000 Ljubljana, Slovenia;; ^e^Research Software Development Group, Research IT Services, University College London, London WC1E 6BT, United Kingdom;; ^f^The Alan Turing Institute, London NW1 2DB, United Kingdom;; ^g^Department of Computer Science, University College London, London WC1E 6BT, United Kingdom;; ^h^School of Engineering, Institute for Multiscale Thermofluids, The University of Edinburgh, Edinburgh EH9 3FD, United Kingdom;; ^i^Department of Computer Science, University of Oxford, Oxford OX1 3QD, United Kingdom;; ^j^Centre de Recerca Matemàtica, 08193 Barcelona, Spain;; ^k^ICREA, Institució Catalana de Recerca i Estudis Avançats, 08010 Barcelona, Spain

**Keywords:** tumor vasculature, hematocrit dynamics, oxygen heterogeneity, anti-angiogenic agents, mathematical modelling

## Abstract

Oxygen heterogeneity in solid tumors is recognized as a limiting factor for therapeutic efficacy. This heterogeneity arises from the abnormal vascular structure of the tumor, but the precise mechanisms linking abnormal structure and compromised oxygen transport are only partially understood. In this paper, we investigate the role that red blood cell (RBC) transport plays in establishing oxygen heterogeneity in tumor tissue. We focus on heterogeneity driven by network effects, which are challenging to observe experimentally due to the reduced fields of view typically considered. Motivated by our findings of abnormal vascular patterns linked to deviations from current RBC transport theory, we calculated average vessel lengths $\bar{L}$ and diameters $\bar{d}$ from tumor allografts of three cancer cell lines and observed a substantial reduction in the ratio $\lambda =\bar{L}/\bar{d}$ compared to physiological conditions. Mathematical modeling reveals that small values of the ratio λ (i.e., λ<6) can bias hematocrit distribution in tumor vascular networks and drive heterogeneous oxygenation of tumor tissue. Finally, we show an increase in the value of λ in tumor vascular networks following treatment with the antiangiogenic cancer agent DC101. Based on our findings, we propose λ as an effective way of monitoring the efficacy of antiangiogenic agents and as a proxy measure of perfusion and oxygenation in tumor tissue undergoing antiangiogenic treatment.

## Significance

Oxygen heterogeneity in solid tumors is recognized as a limiting factor for therapeutic efficacy. This heterogeneity arises from the abnormal tumor vascular structure. We investigate the role that anomalies in red blood cell transport plays in establishing oxygen heterogeneity in tumor tissue. We introduce a metric to characterize tumor vasculature (mean vessel length-to-diameter ratio, λ) and demonstrate how it predicts tissue-oxygen heterogeneity. We also report an increase in λ following treatment with the antiangiogenic agent DC101. Together, we propose λ as an effective way of monitoring the action of antiangiogenic agents and a proxy measure of oxygen heterogeneity in tumor tissue. Unraveling the causal relationship between tumor vascular structure and tissue oxygenation will pave the way for new personalized therapeutic approaches.

Tissue oxygenation plays a crucial role in the growth and response to treatment of cancer. Indeed, well-oxygenated tumor regions respond to radiotherapy better than hypoxic, or oxygen-deficient, regions by a factor of up to three (1, 2). Further, the increased rates of proteomic and genomic modifications and clonal selection associated with anoxia (i.e., total oxygen depletion) endow tumors with more aggressive and metastatic phenotypes (3, 4). Oxygen heterogeneity in solid tumors is commonly attributed to their abnormal vasculature (5, 6). This link is arguably multifactorial, including nonuniform vessel distribution, inefficient vessel organization (e.g., functional shunting), flow fluctuations, and variations in red blood cell (RBC) flux (see ref. 7 for a review). However, the dynamics of abnormal oxygen transport at the whole vascular-network level have not been addressed.

Oxygen is transported through the vasculature by binding to hemoglobin in RBCs (8). Hematocrit, or the volume fraction of RBCs in whole blood, does not distribute uniformly throughout the vasculature (9, 10). At a bifurcation with one afferent and two efferent branches, it is typically assumed that the efferent branch with the highest flow rate will have the highest discharge hematocrit (10, 11) due to, among other features, plasma skimming (PS) caused by the presence of an RBC-depleted layer, or cell-free layer (CFL) (12). In addition, confinement effects can lead to further reduction of the tube hematocrit compared to the discharge value. In this study, we will refer to discharge hematocrit only. How abnormal tumor vascularization impacts hematocrit splitting (HS) at bifurcations and the potential network effects arising have not been addressed.

In this paper, we investigate the role that anomalies in RBC transport play on establishing oxygen heterogeneity in tumor tissue. We focus on heterogeneity driven by network effects, which are difficult to resolve experimentally due to the reduced fields of view typically considered. Live imaging of tumor allografts of three cancer cell lines reveals abnormal morphological vascular patterns, such as short interbifurcation distances and complex topological configurations. Furthermore, detailed numerical simulations describing the transport of RBCs predict deviations from current HS theory in these scenarios. When we extract average vessel lengths $\bar{L}$ and diameters $\bar{d}$ from tumor allografts of three cancer cell lines, we observe a substantial reduction in the ratio $\lambda =\bar{L}/\bar{d}$, an accepted parameter governing CFL dynamics, compared to physiological conditions. In addition, our RBC simulations reveal 1) asymmetric CFL width disruption following a bifurcation; and 2) that the average measured λ value in tumor allografts is too small for the CFL to recover full symmetry between consecutive branching points, leading to uneven hematocrit split in the downstream branching point.

Based on the RBC simulations, we propose an HS rule that accounts for CFL disruption due to pathologically small λ values. We integrate this rule into existing models of tumor blood flow and oxygen transport (13) and observe a hematocrit memory effect leading to hemoconcentration/hemodilution in densely branched vessel networks. As a consequence, the predicted tissue oxygenation is highly heterogeneous and differs markedly from predictions generated by using rules for HS under physiological conditions (e.g., refs. 5 and 14–17). These findings provide a mechanistic explanation for previous reports of hemoconcentration/hemodilution in tumor vasculature (18), plasma flow (19), and well-perfused vessels that are hypoxic (20). Furthermore, previous work has demonstrated that fluctuations in RBC flux in the tumor microvasculature are associated with instabilities in oxygen concentration in the extravascular space (21, 22). However, the mechanism underlying these fluctuations remains unclear. Our findings of hematocrit network effects support the hypothesis that vascular structural instability (associated with vascular remodeling or angiogenesis) can induce long-range changes in RBC flux throughout the network.

Finally, we demonstrate the preclinical relevance of our findings by showing an increase in the average λ value of tumor vascular networks following treatment with the DC101 antiangiogenic cancer agent. Based on our results, we propose λ as an effective way of monitoring the action of antiangiogenic agents and as a proxy measure of oxygen heterogeneity in tumor tissue undergoing antiangiogenic treatment. Future experimental studies should confirm this finding and elucidate its relative importance compared to well-characterized mechanisms of normalization [e.g., permeability reduction or vessel decompression (23)].

## Results

### Average Distance between Vessel Branch Points Is Shorter in Solid Tumors than in Healthy Tissue.

**Fig. 1.**
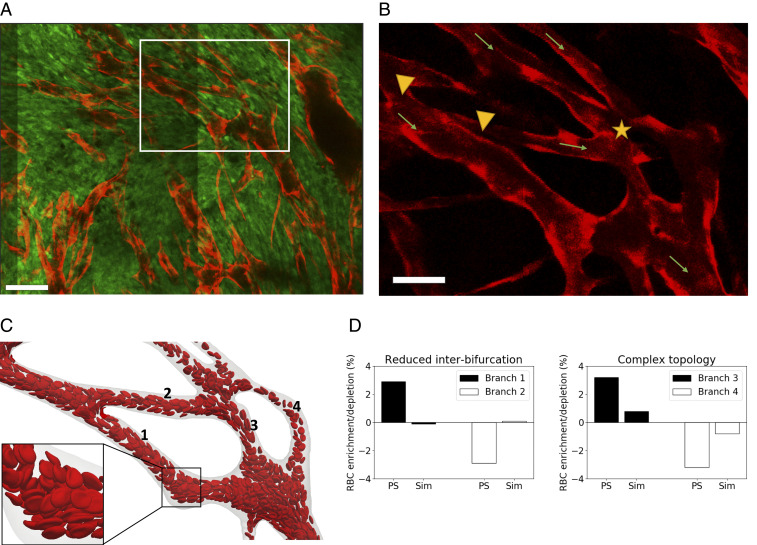
(*A*) Multiphoton image (0.83 μm × 0.83 μm × 3 μm resolution) of a MC38 tumor vessel network obtained via an abdominal imaging window in mouse. Red, endothelial cells; green, tumor cells. White box shows region of interest. (Scale bar, 100 μm.) (*B*) Region of interest showing endothelial cell-staining channel alone. Abnormal vascular morphology: reduced interbifurcation distance (between arrowheads) and complex topology (three- to two-branch merger, star). Arrows indicate direction of flow. (Scale bar, 50 μm.) (*C*) RBC flow simulation in realistic network with vessel identities in computational domain reconstructed from region of interest. (*D*) RBC flow-simulation analysis. RBC enrichment/depletion after branching point. Solid line represents proportional partitioning based on flow rates. Existing PS theory predicts enrichment in higher-flow branch. RBC simulations (Sim) show inversion of effect in reduced interbifurcation case and strong attention in complex topology case. PS theory refers to ref. 11.

We implemented a protocol for in vivo imaging of tumor vasculature (24) and exploited our recently published methods for vessel segmentation (25, 26) and three-dimensional (3D) vascular network reconstruction to characterize the morphology of tumor vasculature. Most analyses of tumor vasculature are based on histological sections that give only partial information about the 3D structure of the entire network. Vascular casts (see ref. 27 for a review) and optical clearance techniques (28) have been used successfully to image the complete tumor vasculature, but they provide information at only one time point. Further, information about vessel perfusion and/or the physiological pressures that drive blood flow are not available. By applying intravital video microscopy to mice in which all of the endothelium is fluorescent, it was possible to image the entire tumor vasculature: Individual vessels could be detected, regardless of their level of perfusion (see *Materials and Methods* for more details). Briefly, tumor allografts of three murine cancer cell lines (i.e., MC38, colorectal carcinoma; B16F10, melanoma; and LLC, Lewis lung carcinoma) were implanted in mice, controlled for size, and imaged through an abdominal window chamber by using a multiphoton microscope over multiple days. The vascular networks in the 3D image stacks were segmented, and the associated network skeletons and vessel diameters were computed (25, 26). Flow direction was determined by tracking the movement of fluorescently labeled RBCs. Upon inspection, the segmented vascular networks presented abnormal morphological vascular patterns, such as short interbifurcation distances, and complex topological configurations, such as three- to two-branch mergers (Fig. 1 *A* and *B*). Motivated by these findings, we exploited recent advances in blood-flow simulation methods by our group and others (29–32) to investigate the link between these abnormal patterns and RBC transport. A computational model of liquid-filled deformable particles (discocytes approximating the shape of an RBC) suspended in an ambient fluid was used to simulate blood flow in a subset of the network, with RBCs inserted at the network inlet and removed at the outlets (see *Materials and Methods* and *SI Appendix* for more details). Our simulations demonstrate that reduced interbifurcation distance can lead to an inversion of HS, as predicted by existing theory (11), where the branch with highest flow receives proportionally fewer RBCs and vice versa (Fig. 1 *C* and *D*). In addition, the same effect is highly attenuated in the complex topology case.

**Fig. 2.**
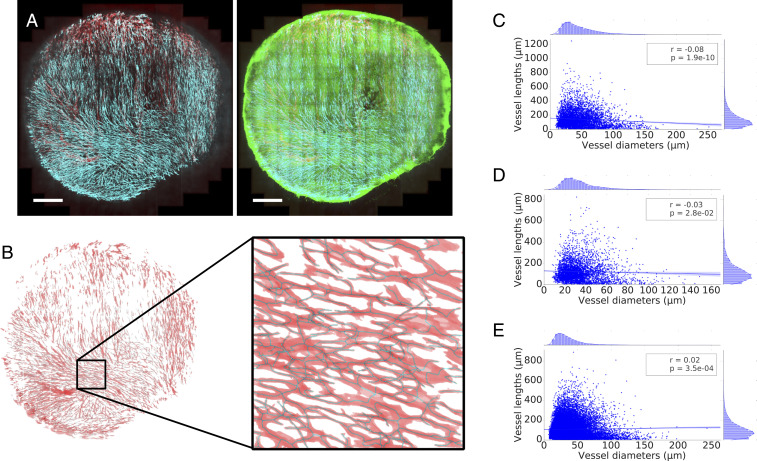
(*A*) Maximum intensity projection of multiphoton image stack of a tumor vessel network obtained via an abdominal imaging window in mouse from the MC38 dataset. Red, perfusion; cyan, endothelial cells; green, GFP tumor cells. (Scale bars: 1 mm.) (*B*) The stack is subsequently segmented and skeletonized, and distributions of vessel diameters and lengths are calculated. Red, vascular network segmentation; dark, skeletonization. Mouse MC38-5 in Table 1. (*C*–*E*) Scatter plots of vessel lengths vs. diameters for different cell lines studied: MC38 (mouse 3 in Table 1) (*C*), B16F10 (mouse 1 in Table 1) (*D*), and LLC (mouse 1 in Table 1) (*E*).

We then asked how prevalent these abnormal vascularization patterns are throughout the tumor networks imaged. Fig. 2 *A* and *B* shows the two-dimensional (2D) maximum projection of an example dataset, along with a 2D projection of its segmentation and a close-in overlaying segmentation and skeletonization. Vessel lengths (L) and diameters (d) in the networks followed a right-skewed distribution resembling a log-normal distribution (Fig. 2 *C*–*E* and Datasets S1–S3). No correlation was found between the variables (Pearson’s $r^{2}<0.04$ for all samples analyzed; Fig. 2 *C*–*E* and *SI Appendix*, Tables S4 and S5).

Table 1 summarizes last-day statistics for all of the experiments and averages per cell line. In the example MC38 dataset from Fig. 2*A*, average vessel length ($\bar{L}$) and diameter ($\bar{d}$) were 143 and 45.5 μm, respectively. We observed how the group average vessel length was 128.6, 125.9, and 108.8 μm for MC38, B16F10, and LLC, respectively. The average diameters were 33, 36.5, and 35.7 μm, respectively, which is within the range described for tumor vasculature (33). In addition, the length-to-diameter ratios (λ) are 4.0,3.4,3.0, respectively, which is substantially smaller than typical λ values reported under physiological conditions in a variety of tissues (*SI Appendix*, Table S1) and representative of the high branching density encountered in tumor vasculature (34) and in line with the observation in Fig. 1*B*.

**Table 1.** Mean branch lengths ($\bar{L}$), mean vessel diameters ($\bar{d}$), and length-to-diameter ratio ($\lambda =\frac{\bar{L}}{\bar{d}}=\frac{\sum L_{i}}{\sum d_{i}}$) measured over all of the imaged vessels in tumor allografts from three murine cancer cell lines

Cell line	MC38							B16F10				LLC
Mouse	1	2	3	4	5	6	Av.	1	2	3	Av.	1
$\bar{L},\mu$m	123.9	129.9	143	112.4	132.1	130.0	128.6	123.2	123.5	131.0	125.9	108.8
$\bar{d},\mu$m	29.3	33.0	45.5	23.9	28.9	37.5	33.0	33.9	34.1	41.6	36.5	35.7
$\lambda =\bar{L}/\bar{d}$	4.2	3.9	3.1	4.7	4.6	3.4	4.0	3.6	3.6	3.1	3.4	3.0

See *SI Appendix*, Tables S4 and S5 for correlation between variables. Av., average.

### PS in Tumor-Like Vasculature Is Biased by History Effects Arising from CFL Dynamics.

Our finding of reduced interbranching point distance in tumor tissue motivated us to investigate a potential causal relationship between the reduction in L and λ and the profoundly abnormal tumor hemodynamics and mass transport patterns described in the literature (35). Of particular interest was establishing whether hemorheological phenomena may contribute to tumor heterogeneity and hypoxia.

The presence of an RBC-depleted region adjacent to the vessel walls (i.e., the CFL) is a key contributor to PS (10–12). Previous studies have shown CFL disruption after microvascular bifurcations and found that the length required for CFL recovery, $l_{r}$, is in the region of 10 vessel diameters (d) for d<40 μm (11), 8 to 15d for d∈[20,24]μm (36), and 25d for d∈[10,100]μm (37). These values are substantially higher than the average λ values given in Table 1, $\lambda <l_{r}$, and, therefore, we expect that, on average, CFL symmetry will not recover between the branching points in the networks under study.

**Fig. 3.**
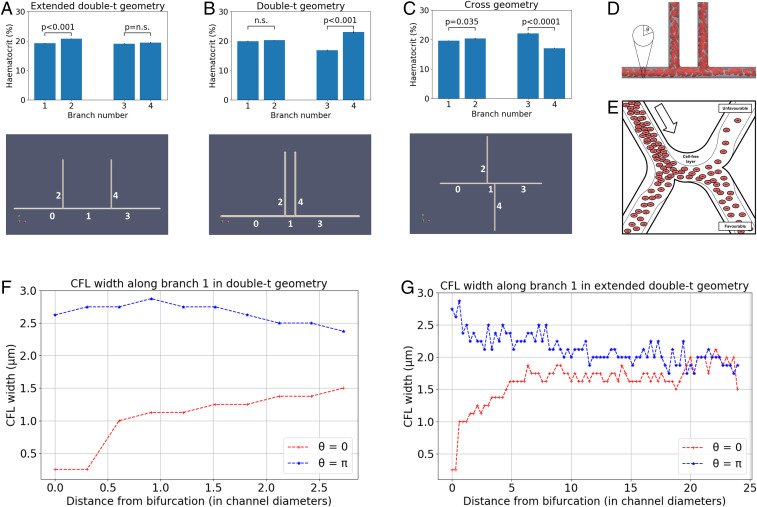
(*A*–*C*) Discharge hematocrit at different geometry branches: extended double-t geometry (*A*), double-t geometry (*B*), and cross geometry (*C*). (*D*) Example simulated RBCs in the double-t geometry: The vessel network is rendered semitransparent in gray, and the RBC membranes are rendered in red suspended in transparent blood plasma. (*E*) Schematic describing the impact of CFL dynamics on hematocrit split. (*F* and *G*) CFL width in opposite sides of channel 1: double-t geometry (*F*) and extended double-t geometry (*G*).

Motivated by these findings, we applied our RBC simulation approach to investigate the link between CFL dynamics and PS in a tumor-inspired 3D microvascular network. Our intention was to understand the extent to which CFL disruption effects arising at a bifurcation affect HS in downstream bifurcations for small interbifurcation distances relevant to tumor vasculature (see *Materials and Methods* for further details). Briefly, we defined a set of networks of 3D cylindrical channels of constant radius, consisting of one main channel with an inlet and an outlet and two side branches, which defined two consecutive bifurcations (Fig. 3). We considered interbifurcation distances of 4 and 25 channel diameters based on our tumor vascular network analysis and the largest of the CFL recovery distances reviewed earlier. We positioned the two side branches on the same side of the main channel or on opposite sides. Flow rates at the network inlet and outlets were configured such that at each bifurcation, flow was split evenly. We performed blood-flow simulations (three runs in each network, with random perturbations in the RBC insertion procedure), and, after the initial transient time required to fully populate the network with RBCs, we quantified discharge hematocrit by an RBC-counting procedure.

Fig. 3 *A* and *B* and Table 2 show how hematocrit split is close to even at bifurcation 1 for all geometries studied, as would be predicted by existing theoretical models of HS. However, different degrees of HS occur at bifurcation 2. In the double-t geometry, we observed hemodilution in branch 3 and hemoconcentration in branch 4 (16.8% vs. 23%, P<0.001; Fig. 3*B*), which we will refer to as the unfavorable and favorable branches. These effects are not statistically significant in the same branches in the extended double-t geometry (19.1% vs. 19.4%, P=0.3; Fig. 3*A*). The hemoconcentration/hemodilution effect is present in the cross geometry, but the branches experiencing it are interchanged (22.1% vs. 17.1%, P<0.001; Fig. 3*C*). In contrast with these results, existing HS theoretical models would predict even HS at bifurcation 2, regardless of the interbifurcation distance, due to the prescribed symmetrical flow and geometry conditions.

**Table 2.** Discharge hematocrit calculated at the different branches of each bifurcation for the extended double-t (EDT) geometry, double-t (DT) geometry, and cross (X) geometry

	Branch 0	Branch 1	Branch 2
Bifurcation 1			
EDT	20.06 (0.03)	19.23 (0.14)	20.80 (0.10)
DT	20.08 (0.06)	19.96 (0.19)	20.26 (0.05)
X	20.06 (0.02)	19.67 (0.09)	20.35 (0.20)
Bifurcation 2			
EDT	19.23 (0.14)	19.05 (0.22)	19.4 (0.23)
DT	19.96 (0.19)	16.83 (0.24)	23.04 (0.46)
X	19.67 (0.09)	22.12 (0.26)	17.09 (0.13)

Values are given as mean (SE) over an ensemble of three simulations with random perturbations in the RBC insertion procedure while the hematocrit at the inlet is held constant.

On closer inspection, the dynamics of the CFL show how, after bifurcation 1, CFL width is initially negligible and rapidly increases on the side of channel 1, leading to the favorable branch (θ=0; Fig. 3*F*). Conversely, CFL width increased after the bifurcation and followed a downward trend in the opposite side (θ=π; Fig. 3*F*). An interbifurcation distance of four diameters is too short for the CFL width to equalize on both sides (Fig. 3*F*). In contrast, CFL width has time to become symmetric on both sides for an interbifurcation distance of 25 diameters (Fig. 3*G*).

Taken together, these results show how CFL asymmetry can cause uneven hematocrit split in bifurcation 2 (Fig. 3*E*), irrespective of branching side—i.e., cross vs. double-t geometry. Our results are consistent with the findings by Pries et al. (11), describing how asymmetry of the hematocrit profile in the feeding vessel of a bifurcation has a significant influence on RBC distribution in the child vessels. In addition, we provide quantitative evidence of how CFL asymmetry may be the main contributing factor.

Interestingly, we observed small, but statistically significant, asymmetries in the hematocrit split in bifurcation 1 in the extended double-t geometry (19.2% vs. 20.8%, P<0.001; Fig. 3*A*) and cross geometry (19.7% vs. 20.4%, P=0.035; Fig. 3*C*), which consistently favored the side branch. We attribute this secondary effect to an asymmetrical streamline split in the chosen geometry, as investigated in ref. 38.

We note that the effects described above depend on the angle between the planes containing the two consecutive bifurcations. Our data suggest that for angles of $\frac{\pi }{2}$ radian, the asymmetric hematocrit split effects will not be observed since the CFL width at $\frac{\pi }{2}$ and $\frac{3\pi }{2}$ remained mostly symmetric (*SI Appendix*, Fig. S2).

### Hematocrit History Effects Lead to Highly Heterogeneous Oxygen Distributions in Solid Tumors.

Existing theoretical models of HS (11, 39–41) do not capture the hemoconcentration/hemodilution effects in realistic and idealized vascular networks presented in Figs. 1 and 3. We hypothesize that this is because they neglect CFL disruption at bifurcations and its impact on subsequent bifurcations. We propose an HS model which accounts for CFL dynamics and show that it predicts history effects in dense networks (see *Materials and Methods* for details and *SI Appendix* for a description of its validation). The model is significantly less computationally intensive to solve than the RBC simulations (see *Materials and Methods* for details).

We used Murray’s law (42) and our experimentally measured values of λ to design a synthetic vessel network comprising consecutive double-t/cross bifurcations (see *SI Appendix*, Fig. S4*A* and *Materials and Methods* for details). Most notably, at all bifurcations, the flow split and the radii of the child vessels were equal, a scenario where existing HS models would predict homogeneous hematocrit throughout the network. We simulated network blood flow using a Poiseuille flow approximation with an HS model originally proposed by Pries et al. (11, 39) (without memory effects) and our model (accounting for memory effects). As for the RBC simulations, differences in hematocrit between child branches emerged after two bifurcations (*SI Appendix*, Fig. S4*C*) and were amplified with increasing vessel-generation number (*SI Appendix*, Fig. S4*D*).

Our model predicts the emergence of a compensatory mechanism in child branches. Increased flow resistance in the branch experiencing hemoconcentration led to partial rerouting of flow in the other branch (*SI Appendix*, Fig. S4*B*). This, in turn, attenuated the hemoconcentration/hemodilution effects previously described due to HS dependence on flow ratios.

**Fig. 4.**
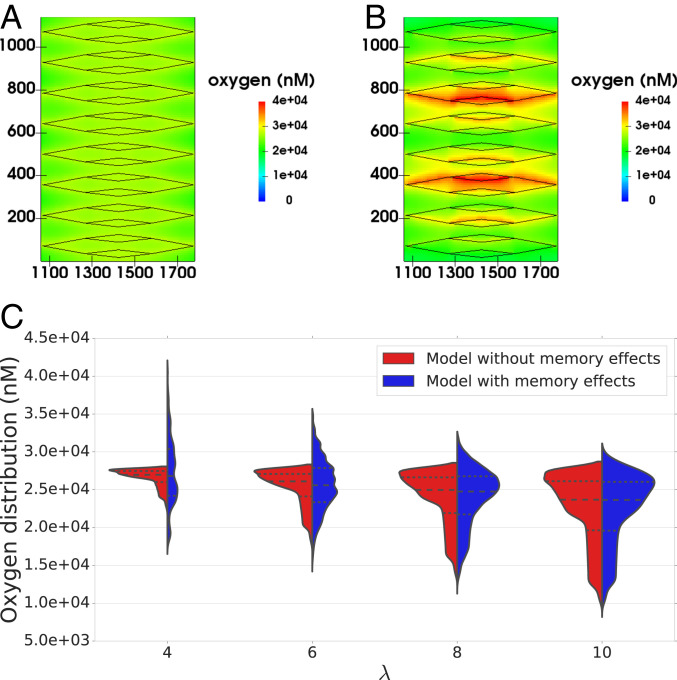
(*A* and *B*) For λ=4.0, the model with memory effects yields more pronounced oxygen heterogeneity than previous theory (11) predicts (i.e. more dispersed oxygen distribution) in *A* (model without memory effects) and *B* (model with memory effects) (spatial scales are in micrometers; vessels are shown in black for reference). (*C*) Violin plots show oxygen distributions for varying λ and the two HS models under consideration. Heterogeneity increases with λ for the model without memory effects, as expected, but the model with memory effects predicts increased heterogeneity for very low λ. The horizontal lines in oxygen distributions in *C* represent 25th, 50th, and 75th percentiles.

We now consider how this memory effect in the hematocrit distribution may affect oxygen distribution in the tissue being perfused by the synthetic network. Following ref. 13 (see *Materials and Methods* for a description of the coupled model), the hematocrit distribution in the network acts as a distributed source term in a reaction–diffusion equation for tissue oxygen. We defined sink terms so that oxygen was consumed at a constant rate everywhere within the tissue. The equation was solved numerically, and oxygen distributions generated by using two HS models (with and without memory effects; *SI Appendix*, Fig. S5 *A* and *B*) were compared for a range of λ values. We focused on the central portion of the network (Fig. 4 *A* and *B*), where the tissue was densely vascularized. The results presented in Fig. 4*C* and *SI Appendix*, Fig. S6 show that for larger values of λ, the differences in the oxygen distribution in the tissue for the two HS models are not statistically significant (e.g., with λ=10, P=0.14). However, as λ decreases, statistically significant differences appear (e.g., with λ=4, P<0.001). Without memory effects, the oxygen distributions become narrower as λ decreases; with memory effects, the oxygen distributions are increasingly wider for λ<10 (*SI Appendix*, Fig. S6*C*), leading to higher dispersion in the distributions for small λ. This is indicative of much more heterogeneous tissue oxygenation.

In the current study, we did not consider other sources of heterogeneity, such as anisotropic transport and heterogeneous consumption of oxygen or other morphological abnormalities in the vascular networks. We hypothesize that their interaction with the hematocrit history effects reported here will further accentuate tissue oxygen heterogeneity.

### Vascular Normalization Therapies Increase λ Ratio in Tumors.

Our findings of reduced λ ratio in tumor vasculature and associated predictions of increased oxygen heterogeneity led us to investigate whether existing vascular normalization therapies modulate this parameter. Previous reports (*SI Appendix*, Table S2) have extensively demonstrated in multiple animal models that antiangiogenic treatment leads to reduction in tumor-vessel diameters. In those studies that analyze vessel length and diameter posttreatment, vessel length either remains unchanged or decreases to a lesser extent than vessel diameter. These findings indicate an increase in λ ratio posttreatment. Furthermore, Kamoun et al. (43) also reported a reduction in tumor hemoconcentration posttreatement, which suggests an in vivo link between an increase in λ, hematocrit normalization, and oxygen-transport homogenization.

**Fig. 5.**
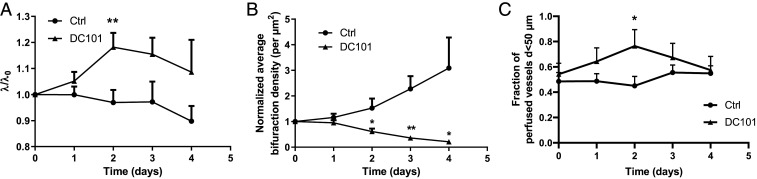
Vascular phenotypes in MC38 tumors over time following DC101 treatment compared with control (*n* = 5). DC101 λ ratio raw data are given in *SI Appendix*, Table S3. (*A*) λ ratio. (*B*) Bifurcation density. (*C*) Fraction of perfused vessels. Plots show mean (solid markers) and SE (error bars). *t* test for statistical significance, *P<0.05; **P<0.01.

We validated these results in our animal model by calculating the λ ratio following DC101 treatment (see *Materials and Methods* for details). Our results indicated that in the first 2 d posttreatment, λ increases significantly and then starts to decrease, matching the control trend (Fig. 5*A*, *SI Appendix*, Table S3, and Dataset S4). This change is explained by a linear increase in vessel length immediately after treatment (absent in the control group), which is compensated after 2 d by an increase at a higher rate in vessel diameter (comparable to the control group) (*SI Appendix*, Fig. S7), and associated with a decrease in bifurcation density, as would be expected from impaired angiogenesis and vessel pruning (Fig. 5*B* and *SI Appendix*, Fig. S7). Our results also demonstrate that, for vessels with diameters less than 50 μm, the fraction of perfused vessels increases after DC101 treatment, reaching a maximum of 63% relative increase compared to control after 2 d, before decreasing to match the controls after 3 d (Fig. 5*C*). We note that the proportion of vessels with diameter less than 50 μm is identical between groups, regardless of perfusion (*SI Appendix*, Fig. S7). Interestingly, the time window in which perfusion peaks coincides with the largest posttreatment increase in λ. However, the structural changes persist beyond this window, which would suggest that improvements in oxygen-transport homogeneity can be independent of perfusion.

## Discussion

Hypoxia compromises the response of many tumors to treatments such as radiotherapy, chemotherapy, and immunotherapy. Dominant causative factors for hypoxia associated with the structure and function of the tumor vasculature include inefficient vessel organization and inadequate flow regulation. Motivated by morphological analyses of vascular networks from different tumor types and detailed computer simulations of RBC transport through realistic and synthetic networks, we have proposed a rheological mechanism for tumor hypoxia.

We analyzed vascular networks from murine MC38, B16F10, and LLC tumor allografts. Upon inspection, the segmented vascular networks presented abnormal morphological patterns, such as reduced interbifurcation distance and branching points with topological configurations different from the typical diverging/converging bifurcations. Detailed computer simulations of RBC transport uncovered a link between these morphological anomalies and strong deviations from the current HS theory, including inversion of HS [a phenomenon that had only been observed in vitro (44, 45)]. For each vessel segment within each network, we calculated the metric λ, which is the ratio of its length and diameter and has been shown to control CFL dynamics. Average λ values for the three tumor cell lines were similar in magnitude ($\bar{\lambda }\in \left[3,4.2\right]$) and severalfold smaller than values from a range of healthy tissues ($\bar{\lambda }\in \left[9.5,70\right]$). Our RBC simulations confirmed previous reports of transient alterations in the CFL downstream of network bifurcations [e.g., asymmetries in the cross-sectional hematocrit profile following a bifurcation (46, 47) and the temporal dynamics governing its axisymmetry recovery (37)]. Further, for the λ values measured in our tumors and the capillary number considered in our simulations, the CFL did not become symmetric between consecutive branching points. This bias is amplified across branching points and drives hemoconcentration/hemodilution at the network level. Based on these findings, we developed a rule for HS at vessel bifurcations that accounts for CFL disruption due to abnormally short vessel segments and validated it against our fully resolved RBC simulations. We then used our existing oxygen-transport model (13) to demonstrate that this hematocrit memory effect can generate heterogeneous oxygen distributions in tissues perfused by highly branched vascular networks and that the network metric λ controls the extent of this heterogeneity. Finally, we reported an increase in the average λ value of tumor vascular networks following treatment with the DC101 antiangiogenic cancer agent.

The implications of our findings are multiple. We have introduced a simple metric to characterize tumor vasculature based on the mean length-to-diameter ratio of vessel segments (λ) and demonstrated how it controls oxygen heterogeneity in a synthetic, densely vascularized tissue model. Our findings, of structurally induced hemodilution in vascular networks with low λ values, provide a mechanistic explanation for experimental observations of hemodilution in tumor vascular networks (18), plasma flow (19), and the existence of well-perfused vessels that are hypoxic (20). Furthermore, these findings support the hypothesis that vascular structural instability (associated with vascular remodeling or angiogenesis) leads to the fluctuations in RBC flux previously linked with cycling hypoxia (21, 22). We conclude that vessel perfusion is a poor surrogate for oxygenation in tissue perfused by vascular networks with low λ values. Further, predictions of tissue oxygenation based on diffusion-dominated oxygen transport (e.g., refs. 5 and (14)–(17)) may be inaccurate if they neglect heterogeneity in the hematocrit distribution of the vessel network.

Finally, antiangiogenic drugs have been shown to generate transient periods of heightened homogeneous tissue oxygenation, due to improved restructuring and reduced permeability of tumor vessels (48). This phenomenon, termed “vascular normalization” (6), can correct the deficient transport capabilities of tumor vasculature and homogenize drug and oxygen coverage, and, thereby, improve radiotherapy and chemotherapy effectiveness (2). Our results demonstrate an increase in the average λ value of tumor vascular networks posttreatment with antiangiogenic drugs and predict that this would lead to a less heterogeneous hematocrit distribution and more uniform intratumoral oxygenation potentially independent of perfusion. Based on our findings, we propose λ as an effective way of monitoring the action of antiangiogenic agents. Further experimental work, quantifying hematocrit network dynamics before and after antiangiogenic treatment, is needed to test this prediction and elucidate its importance in comparison with established mechanisms of normalization [e.g., permeability reduction, vessel decompression (23)]. If confirmed, this finding would provide a theoretical foundation for the development of therapeutic approaches for the normalization of tumor oxygenation involving the administration of agents that target λ and, therefore, homogenize hematocrit and tissue oxygenation.

## Materials and Methods

### Tumor Allograft Model and Abdominal Imaging Window Protocol.

The abdominal window chamber was surgically implanted in transgenic mice on a C57Bl/6 background that had expression of red fluorescent protein tdTomato only in endothelial cells. The murine colon adenocarcinoma (MC38), murine melanoma (B16F10), and murine LLC tumors with expression of green fluorescent protein (GFP) in the cytoplasm were induced by injecting 5 μL of dense cell suspension in a 50/50 mixture of saline and Matrigel (Corning). For DC101 treatment, mice bearing MC38 tumors were treated with anti-mouse VEGFR2 antibody (clone DC101, 500 μg/dose, 27 mg/kg; Bio Cell) injected intraperitoneally on the first and fourth day of imaging. Prior to imaging, we intravenously injected 100 μL of Tracker 705 Vascular Labels (Thermo Fisher Scientific), which is a blood-pool-based labeling agent, thus allowing us to determine whether vessels were perfused or not.

The direction of blood flow in the vessels was determined by tracking the movement of fluorescently labeled RBCs. RBCs were isolated from blood taken from an anesthetized donor mouse via cardiac puncture. RBCs were washed twice in phosphate-buffered saline (PBS) and then labeled with DiD Cell-Labeling Solution (Thermo Fisher). The labeled RBCs were then washed twice again, resuspended in 1 mL of PBS, and then 100 μL of the resuspended labeled RBCs was injected via a tail vein prior to imaging. Isoflurane inhalation anesthesia was used throughout the imaging; mice were kept on a heated stage and in a heated chamber, and their breathing rate was monitored.

Tumor images were acquired with a Zeiss LSM 880 microscope (Carl Zeiss AG), connected to a Mai-Tai tunable laser (Newport Spectra Physics). We used an excitation wavelength of 940 nm, and the emitted light was collected with Gallium Arsenide Phosphide detectors through a 524- to 546-nm bandpass filter for GFP and a 562.5- to 587.5-nm bandpass filter for tdTomato and with a multialkali photomultiplier tube detector through a 670- to 760-nm bandpass filter for Tracker 705 and DiD. A 20× water-immersion objective with numerical aperture of 1.0 was used to acquire a Zstacks-TileScan with dimensions of 512×512 pixels in x and y and approximately 70 planes in z. Voxel size was 3 to 5 μm in the z direction and 0.83 μm × 0.83 μm in the x–y plane. Each tumor was covered by approximately 100 tiles. The morphological characteristics of tumor vasculature were obtained from the acquired images as described (25, 26). All animal studies were performed in accordance with the Animals Scientific Procedures Act of 1986 (UK) and Committee on the Ethics of Animal Experiments of the University of Oxford.

### RBC Simulations in Realistic and Synthetic Capillary Networks.

We created a realistic network of tumor capillaries (*SI Appendix*, Fig. S1) based on the images in Fig. 1 and a described protocol (49, 50). Flow direction at the boundaries was assigned in agreement with experimental observations of fluorescent RBC tracking. The inlets in the domain were connected together to facilitate definition of flow and RBC boundary conditions (and similarly with the outlets).

For our synthetic network simulations, we defined a set of networks of cylindrical channels of diameter d. An inlet channel of length 25d (channel 0) bifurcated into two channels of length δ and 25d at π and π/2 radians clockwise, respectively (channels 1 and 2). Channel 1 bifurcated into two channels of length 25d at π and α radians clockwise, respectively (channels 3 and 4). We considered the following configurations (Fig. 3): double-t geometry (δ=4d,α=π/2), cross geometry (δ=4d,α=3π/2), and extended double-t geometry (δ=25d,α=π/2). Interbifurcation distance δ was calculated between the centerlines of channels 2 and 4 for consistency with our experimental morphological characterization.

A model of liquid-filled elastic membranes (discocytes of 8-μm diameter approximating the shape of an RBC) suspended in an ambient fluid was used to simulate blood flow in the networks. We used the fluid-structure interaction algorithm presented and validated by Krüger et al. (51), which is based on coupling the lattice Boltzmann method (LBM), finite element method, and immerse boundary method. The discocyte membranes were discretized into 500 triangles, which imposed a voxel size of 0.8 μm on the regular grid used in the LBM simulation. The mechanical properties of the membrane were defined to achieve a capillary number (i.e., the ratio of viscous fluid stress acting on the membrane and a characteristic elastic membrane stress) of 0.1 in channel 0. The coupled algorithm is implemented in the HemeLB blood flow simulation software (49, 52) (http://ccs.chem.ucl.ac.uk/hemelb). Simulations ran on up to 456 cores of the ARCHER supercomputer, taking 11 to 32 h. See *SI Appendix* for full details.

A constant flow rate of $Q_{0}={\bar{v}}_{inlet}\pi d^{2}/4$ and a procedure for RBC insertion with discharge hematocrit $H_{inlet}$ was imposed at the network inlet. In the synthetic networks, the outlet flow rates were set to $Q_{2}=Q_{0}/2$ and $Q_{3,4}=Q_{0}/4$ to ensure an equal flow split at each bifurcation. RBCs were removed from the computational domain when they reached the end of any outlet channel. *SI Appendix*, Table S6 summarizes the key parameters in the model. We performed blood-flow simulations (in the synthetic networks, three runs in each network, with random perturbations in the RBC insertion procedure), and, after the initial transient required to fully populate the network with RBCs, we quantified hematocrit by an RBC-counting procedure.

### Hybrid Model for Tissue Oxygen Perfusion that Accounts for History Effects in Vascular Networks.

We first explain how our vascular networks are designed. Then, we describe how blood flow and hematocrit are determined. Next, we introduce the HS models and explain how CFL memory effects are incorporated and the resulting flow problem solved. We conclude by describing how the resulting hematocrit distribution is used to calculate oxygen perfusion in the surrounding tissue. The basic steps of our method are summarized in the flowchart in *SI Appendix*, Fig. S3.

#### Synthetic network design.

Our networks have one inlet vessel (with imposed blood pressure and hematocrit; we call this generation 0), which splits into two child vessels (generation 1), which then split into two child vessels (generation 2), and so on until a prescribed (finite) number of generations is reached. This defines a sequence of consecutive double-t/cross bifurcations. Thereafter, the vessels converge symmetrically in pairs until a single outlet vessel is obtained (with imposed blood pressure). At every bifurcation, the diameters of the two child vessels are assumed to be equal and determined by appealing to Murray’s law (42). Using the same vessel diameters in all simulations, we varied vessel lengths, so that for all vessels in the network, the lengths equaled the product of λ (which is fixed for a given network) and the vessel diameter. We focused on λ-values in the range measured in our tumors. We chose a synthetic network so that only λ-related effects (and not other morphological network characteristics) contributed to hemoconcentration/hemodilution. In future work, we will investigate the combined effect on tissue oxygenation of the HS model with memory effects and other tumor vascular characteristics.

#### Blood flow and HS.

##### Network flow problem.

Tissue oxygenation depends on the hematocrit distribution in the vessel network perfusing the tissue. The hematocrit distribution depends on the blood-flow rates. These rates are determined by analogy with Ohm’s law for electric circuits, with the resistance to flow depending on the local hematocrit via the Fahraeus–Lindquist effect (for details, see *SI Appendix* and ref. 53). The flow rates and hematocrit are coupled. We impose conservation of RBCs at all network nodes.* An HS rule must then be imposed at all diverging bifurcations.

##### HS model without memory effects.

The empirical HS model proposed by Pries et al. (11, 54) states that the volume fraction of RBCs entering a particular branch $FQ_{E}$ depends on the fraction of the total blood flow entering that branch $FQ_{B}$ as follows:

$$\text{logit}\left(FQ_{E}\right)=A+B\text{logit}\left(\frac{FQ_{B}-X_{0}}{1-2X_{0}}\right),$$ [1]

where logit(x)=ln(x/(1−x)), B serves as a fitting parameter for the nonlinear relationship between $FQ_{E}$ and $FQ_{B}$, and A introduces asymmetry between the child branches (note that for an equal flow split $FQ_{B}=0.5$, A ≠ 0 yields uneven splitting of hematocrit). Finally, $X_{0}$ is the minimum flow fraction needed for RBCs to enter a particular branch (for lower-flow fractions, no RBCs will enter)^†^ ; the term $\left(1-2X_{0}\right)$ reflects the fact that the CFL exists in both child vessels (*SI Appendix*, Fig. S8*A*).

##### HS model with memory effects.

We account for the effects of CFL disruption and recovery by modifying the parameters A and $X_{0}$ (as already observed in ref. 11). For simplicity, and in the absence of suitable data, we assume that the parameter B is the same in both child branches. If $X_{0,f}$ ($A_{f}$) and $X_{0,u}$ ($A_{u}$) denote the values of $X_{0}$ (A) in the favorable and unfavorable child branches (Fig. 3*E*), then our model of HS can be written as:

$$\text{logit}\left(FQ_{E,f}\right)=A_{f}+B\text{logit}\left(\frac{FQ_{B,f}-X_{0,f}}{1-X_{0,u}-X_{0,f}}\right),$$ [2]

where subscripts _f_ and _u_ relate to favorable and unfavorable branches, respectively (see *SI Appendix*, Fig. S8*A* for a graphical depiction). It is possible to rewrite Eq. **2** in terms of the suspension flow rates $Q\equiv Q_{B}$ and hematocrit levels H of the favorable f, unfavorable u, and parent P vessels as (for details, see *SI Appendix*):

$$\frac{H_{f}}{H_{u}}=e^{A_{f}}{\left(\frac{Q_{f}-X_{0,f}Q_{P}}{Q_{u}-X_{0,u}Q_{P}}\right)}^{B}\frac{Q_{u}}{Q_{f}}.$$ [3]

This formulation of our HS model facilitates comparison with other HS models (40, 41, 55). Functional forms for $A_{f}$, $X_{0,f}$ and $X_{0,u}$ are based on our RBC simulation results and the existing literature (*SI Appendix*). We used an iterative scheme (as in ref. 40) to determine the flow rates and hematocrit in a given network.

#### Calculating the tissue oxygen distribution.

We embedded the vessel network in a rectangular tissue domain. A steady-state reaction–diffusion equation models the tissue oxygen distribution, with source terms at vessel network locations proportional to the hematocrit there, and sink terms proportional to the local oxygen concentration modeling oxygen consumption by the tissue. This equation was solved numerically by using Microvessel Chaste (see ref. 13 and *SI Appendix* for details). In order to highlight the influence of HS on tissue oxygen, we focused on the central 25% of the domain which is well-perfused and ignored the avascular corner regions (*SI Appendix*, Fig. S5 *A* and *B*).

## Supplementary Material

Supplementary File

Supplementary File

Supplementary File

Supplementary File

Supplementary File

## Data Availability

The source code for the RBC simulations is available at http://ccs.chem.ucl.ac.uk/hemelb. The source code for the oxygen perfusion simulations is available at https://github.com/jmsgrogan/MicrovesselChaste and https://github.com/chaste/chaste. All other data supporting the findings of this study are available within the paper, *SI Appendix*, and Datasets S1–S4.

## Footnotes

*$\underset{i}{\sum }{\tilde{Q}}_{i}H_{i}=0$, where we sum over all vessels i meeting at a given node with hematocrit $H_{i}$ and signed flow rates ${\tilde{Q}}_{i}$ (of magnitude $Q_{i}$).

^†^The dependences of A, B, and $X_{0}$ on the diameters of the participating vessels and on the parent vessel hematocrit are described in *SI Appendix*; refs. 14–16.
