# Supplementary material for: Abnormal morphology biases hematocrit distribution in tumor vasculature and contributes to heterogeneity in tissue oxygenation
Source: Proc Natl Acad Sci U S A. 2020 Oct 27;117(45):27811–9. doi: 10.1073/pnas.2007770117 (PMC7668105; doi:10.1073/pnas.2007770117)
Supplement: Supplementary File [file pnas.2007770117.sapp.pdf]

PNAS
www.pnas.org

Supplementary Information for

Abnormal morphology biases haematocrit distribution in tumour vasculature and contributes to heterogeneity in tissue oxygenation

Miguel O. Bernabeu, Jakub Köry, James A. Grogan, Bostjan Markelc, Albert Beardo, Mayeul d'Avezac, Romain Enjalbert, Jakob Kaeppler, Nicholas Daly, James Hetherington, Timm Krüger, Philip K. Maini, Joe M. Pitt-Francis, Ruth J. Muschel, Tomás Alarcón and Helen M. Byrne

Miguel O. Bernabeu, Helen M. Byrne.
E-mail: miguel.bernabeu@ed.ac.uk, helen.byrne@maths.ox.ac.uk

This PDF file includes:

Supplementary text
Figs. S1 to S8
Tables S1 to S9
SI References

Supporting Information Text

The Supplementary Information is organised as follows. First, we provide experimental evidence which supports the findings that vessel lengths and diameters are uncorrelated in tumour environments. Next, we describe the fluid structure interaction (FSI) algorithm used for the red blood cell (RBC) simulations and the method used to calculate the width of the cell free layer (CFL). Next, we present our hybrid model of tissue perfusion and introduce our new haematocrtic splitting (HS) model. Finally, we comment on the higher mean oxygen values predicted by our oxygen perfusion model for small λ values.

Vessel lengths and diameters in tumour microvasculature are uncorrelated. In Supplementary Tables S4 and S5 we list Pearson's r-values quantifying the correlation between vessel lengths, L, and diameters, d,

$$\rho_{L,d} = \frac{\mathrm{cov}(L,d)}{\sigma_L \sigma_d} \ , \tag{1}$$

where $\mathrm{cov}(i,j)$ is the covariance of two variables and σ_i is the standard deviation of variable i, for the three tumour cell lines used in our experiments. Results are presented for each mouse and each scan. Day 0 was chosen as the day when the tumour vascular network appeared to be fully formed. This typically occurred approximately 8 days after tumour induction, when the tumour size was approximately 4 mm in diameter. We note also that the duration of the observation period is cell-line specific; some tumours grew faster than others and, as a result, soon started pushing on the window, and in such cases the animal had to be culled as per licence limitations. The Pearson's r-values are too low to conclude that a correlation exists between L and d in the tumour vascular networks studied.

Red blood cell suspension model. The lattice Boltzmann method (LBM) numerically approximates the solution of the Navier-Stokes equations for a weakly compressible Newtonian fluid discretised on a regular lattice. We employ the D3Q19 lattice, the Bhatnagar–Gross–Krook collision operator extended with the Guo forcing scheme (1), the Bouzidi-Firdaouss-Lallemand (BFL) implementation of the no-slip boundary condition at the walls (2), and the Ladd implementation of the velocity boundary condition for open boundaries (3). These methods have been extensively used and analysed in the literature (see (4, 5) for a detailed description).

The RBC membrane is modelled as a hyperelastic, isotropic and homogeneous material, following the model described in (6). The total membrane energy W is defined by $W = W^S + W^B + W^A + W^V$, where the superscripts denote energy contributions due to strain, bending, area and volume. We employ the surface strain energy density w^S proposed by Skalak *et al.* (7):

$$w^S = \frac{\kappa_s}{12}\left(I_1^2 + 2I_1 - 2I_2\right) + \frac{\kappa_\alpha}{12} I_2^2 \ , \tag{2}$$

$$I_1 = \lambda_1^2 + \lambda_2^2 - 2 \ , \tag{3}$$

$$I_2 = \lambda_1^2 \lambda_2^2 - 1 \ , \tag{4}$$

where κ_s and κ_α are the shear and dilation moduli, λ_1, λ_2 are the local principal in-plane stretch ratios (see (8) for calculation procedure), and $W^S = \int dA \ w^S$. The shape of the discocyte membrane is approximated by a number N_f of flat triangular faces, and W^S is numerically calculated based on a finite element method (FEM) approach as

$$W^S = \sum_{j=1}^{N_f} A_j^{(0)} w_j^s \ , \tag{5}$$

where $A_j^{(0)}$ is the undeformed area of face j. The bending energy of the RBC membrane is numerically calculated as

$$W^B = \sqrt{3}\kappa_B \sum_{\langle i,j \rangle} \left(\theta_{i,j} - \theta_{i,j}^{(0)}\right)^2 \ , \tag{6}$$

where κ_B is the bending modulus, $\theta_{i,j}$ is the angle between the normals of two neighbouring faces i and j, and $\theta_{i,j}^{(0)}$ is the same angle for the undeformed membrane. Finally, we penalise deviations of the total membrane surface area and volume by defining two additional energy contributions:

$$W^A = \frac{\kappa_A}{2} \frac{\left(A - A^{(0)}\right)^2}{A^{(0)}} \ , \tag{7}$$

$$W^V = \frac{\kappa_V}{2} \frac{\left(V - V^{(0)}\right)^2}{V^{(0)}} \ , \tag{8}$$

where κ_A, κ_V are the surface area and volume moduli, A and $A^{(0)}$ are the current and undeformed membrane surface areas, and similarly with V. The principle of virtual work yields the force acting on each membrane vertex i at position $\vec{x}_i$ through

$$\vec{F}_i = -\frac{\partial W(\{\vec{x}_i\})}{\partial \vec{x}_i}. \tag{9}$$

The immersed boundary method (9) is used to couple the fluid and membrane dynamics. The fluid velocity is interpolated at the positions $\vec{x}_i$ of the RBC mesh vertices, and a forward-Euler scheme is used to advect the vertices to satisfy the no-slip condition. The vertex forces $\vec{F}_i$ are spread to the lattice where they are used as input to the forcing term in the LBM, which ensures local momentum exchange between the membrane and the fluid. See (6) for a detailed numerical analysis of the algorithm.

The RBC model contains five parameters (κ_s, κ_α, κ_B, κ_A, and κ_V). While κ_s and κ_B are known from experiments (see review in (10)), the exact values of the three remaining parameters are chosen to ensure that local area, total surface area and volume drift are constrained within a few percent and simulations are stable (see analysis is (6, 8)). Table S7 summarises all the parameters in the model.

CFL width calculation. To calculate the CFL width in channel 1 of the domains in Figure 3, let us consider a vessel cross-section of diameter d at distance l downstream from the first bifurcation in the network. The RBC density, $\phi(r, \theta, l, t)$, is 1 if there is a RBC at time t occupying the point with radial coordinate r and angular coordinate θ of the cross-section and 0 otherwise. The average RBC density flux $\Phi(l)$ going through the cross-section is

$$\Phi(l) = \frac{1}{N}\sum_{i=1}^{N}\int_0^{2\pi} d\theta \int_0^{d/2} r dr \phi(r, \theta, l, t_i)\mathbf{v}(r, \theta, l, t_i) \cdot \mathbf{n} \,,$$

where $\mathbf{v}$ is the fluid velocity, $\mathbf{n}$ is the cross-section normal vector and N is the number of simulation time steps in the average (0.5 s of real time simulation sampled every 0.0215 s, $N = 23$, in our case).

We define $\chi = 0.01$ as the average fraction of RBC density flux crossing the CFL. Now we are able to numerically determine the local CFL width $W(l, \theta)$: consider a 2D-cone centered and contained in the cross-section with orientation θ and size $\Delta\theta = \pi/2$. The width $W(l, \theta)$ is the distance such that

$$\chi = \frac{4}{\Phi N}\sum_{i=1}^{N}\int_{\theta-\Delta\theta/2}^{\theta+\Delta\theta/2} d\theta' \int_{d/2-W}^{d/2} r dr \phi(r, \theta', l, t_i)\mathbf{v}(r, \theta', l, t_i) \cdot \mathbf{n} \,.$$

Since we are only interested in the spatial evolution of the CFL, the specific value of χ used in the definition is arbitrary. The choice of χ will change the width of the CFL after symmetry recovery, but it will not affect the local characterisation of the CFL spatial evolution after a bifurcation. For example, for any value of χ, the CFL recovery distance can be calculated as the shortest distance l for which the CFL width W do not depend on coordinate θ.

Hybrid model of oxygen transport in vascularised tissue.

Choice of vessel diameters and branching angles in vascular networks. In the branched networks used, we fix the diameter of the inlet vessel so that $d_{inlet} = 100$ μm. The diameters of the two child vessels (d_α and d_β) are assumed to be equal and determined from the diameter of the parent vessel (d_P) via Murray's law (11) so that:

$$d_P^3 = d_\alpha^3 + d_\beta^3 \equiv 2d_\alpha^3,$$

in which case

$$d_\alpha = \frac{d_P}{2^{\frac{1}{3}}}.$$

Since the network is symmetric about its central axis, vessels on the converging side have the same diameters as those of the same generation on the diverging side (see Supplementary Figure S4a). For all simulations the networks have 6 generations of vessels. The length L of a vessel segment in a given network is related to its diameter d via $L = \lambda d$, where the positive constant λ is network-specific.

For complete specification of the network geometry, in two-dimensional Cartesian geometry, it remains to embed the network in a spatial domain. This is achieved by specifying either the branching angles, or (equivalently) the lengths of the projections of the vessels on the y axis. Denoting by L_1^{vert} the length of the projection of a vessel of generation 1, the lengths of the projections of vessels of generation $i > 1$ are given by $L_i^{vert} = \frac{1}{2}L_{i-1}^{vert}$. As a result, the vertical size of the domain will not exceed $4L_1^{vert}$ for any number of generations. Finally, we require $L_1^{vert} < L_1 =$ length of vessels of generation 1. In our simulations, we fix $L_1^{vert} = 0.9L_1$ to ensure adequate spatial extent in the y-direction.

Poiseuille's law and the Fahraeus-Lindquist effect. We simulate flow in the branched networks by following the approach of Pries *et al.* (12). For blood vessels of length L and diameter d, we assume Poiseuille's law

$$Q = \frac{\pi}{128}\frac{\Delta p d^4}{L\mu}, \qquad [10]$$

where Q is the vessel flow rate, Δp is the pressure drop along the vessel and μ is the effective viscosity of blood (13). Following (14) we assume that the blood viscosity depends on vessel diameter and haematocrit via the empirical relationship:

$$\mu = \mu_p\left[1 + (\mu_{45} - 1)\frac{(1-H)^C - 1}{(1-0.45)^C - 1}\left(\frac{d}{d-1.1}\right)^2\right]\left[\frac{d}{d-1.1}\right]^2,$$

where μ_p is the plasma viscosity, H is the vessel discharge haematocrit,

$$\mu_{45} = 6e^{-0.085d} + 3.2 - 2.44e^{-0.06d^{0.645}}$$

and

$$C = \left(0.8 + e^{-0.075d}\right)\left(-1 + \frac{1}{1+10^{-11}d^{12}}\right) + \frac{1}{1+10^{-11}d^{12}}.$$

Introducing signed flow rates $\tilde{Q}_i$ for the sake of brevity, we impose conservation of blood and haematocrit at each network bifurcation, so that

$$\sum_i \tilde{Q}_i = 0, \qquad [11]$$

and

$$\sum_i \tilde{Q}_i H_i = 0. \qquad [12]$$

In Eq. (11) and Eq. (12) we sum over the three vessels that meet at that bifurcation. At diverging bifurcations, we impose a HS rule: we use (1) from the main text when CFL memory effects are neglected and (2) from the main text when they are included. Denoting by N_B the number of network bifurcations and N_V the number of vessels, we have N_B unknown pressures P, N_V unknown flow rates Q and $N_V - 1$ unknown haematocrit levels (the inlet haematocrit being prescribed) - altogether $N_B + 2N_V - 1$ unknowns. At the same time, we impose Poiseuille's law (Eq. (10)) for every vessel (N_V times), conservation of blood (Eq. (11)) and haematocrit (Eq. (12)) at every bifurcation node (N_B times), and an HS rule at all diverging bifurcations ($N_B/2$ times), yielding a total of $N_V + 5N_B/2$ algebraic equations. Since every bifurcation connects 3 vessels, we have $N_V = (3N_B + 2)/2$, where every vessel is counted twice, except for the inlet and outlet vessels (+2 in the numerator). From this, it follows that the number of equations ($N_V + 5N_B/2$) equals the number of unknowns ($N_B + 2N_V - 1$).

Oxygen distribution in tissue. In this section, we determine the oxygen concentration c in the tissue. Following (15), we assume that the dominant processes governing its distribution are delivery from the vessel network (via one-way coupling with Eq. (12) and the haematocrit models (1) or (2), *i.e.* c depends on H_l but not vice versa), diffusive transport through the tissue, and consumption by cells in the tissue. We focus on the long time behaviour and, therefore, adopt a quasi-steady state approximation (16)

$$\underbrace{D\nabla^2 c}_{\substack{\text{diffusive transport}\\ \text{through the tissue}}} \underbrace{+ \pi d_l \gamma \left(\frac{\beta_{ref}}{H_{ref}} H_l - c\right)\delta_{network}}_{\substack{\text{delivery from the}\\ \text{blood vessels}}} - \underbrace{\kappa c}_{\substack{\text{consumption by}\\ \text{the tissue}}} = 0\,. \qquad [13]$$

In Eq. (13), the positive constants D, γ and κ represent the diffusion coefficient for oxygen in the tissue, the vessel permeability to oxygen, and the rate at which it is consumed by cells in the tissue. The vessel network is represented by a collection of Dirac point sources $\delta_{network}$ where

$$\delta_{network}(\boldsymbol{x}) = \begin{cases} 1 & \text{if vessel is located at } \boldsymbol{x} \\ 0 & \text{otherwise} \end{cases}$$

and for any $\boldsymbol{x}$ satisfying $\delta_{network}(\boldsymbol{x}) = 1$, d_l and H_l are the diameter and haematocrit of the vessel at that location (where the latter has been calculated as described in the previous section). The constant β_{ref} represents the oxygen concentration of a reference vessel containing haematocrit H_{ref} (here we fix $H_{ref} = 0.45$, the inlet haematocrit) and we suppose that the oxygen concentration of a vessel with haematocrit H_l is $\beta_{ref} H_l / H_{ref}$. In Eq. (13) we assume that the oxygen is supplied by vessels to the tissue at a rate which is proportional to their circumference πd_l, the vessel permeability γ, and $\beta_{ref} H_l / H_{ref} - c$. Finally, we have $\beta_{ref} = c_{stp} p_{ref} \alpha_{eff}$, where c_{stp} denotes an ideal gas concentration at standard temperature and pressure, p_{ref} denotes the reference partial pressure at the inlet vessel, and α_{eff} denotes the volumetric oxygen solubility (17). A summary of the parameter values used to solve Eq. (13) is presented in Supplementary Table S8.

Derivation of, and justification for, the HS model with CFL memory.

Parameter dependencies in HS model without memory from (18). The dependencies of the parameters A, B and X_0 (see (1) from the main text) on the diameters of the parent and child vessels (d_P, d_α and d_β, respectively), and the discharge haematocrit H_P in the parent vessel were first introduced in (12, 19) and later adjusted in (18) to achieve a better approximation under extreme combinations of d_α, d_β, d_P and H_P. We will use the functional forms from (18), which read

$$A = -13.29\left[\left(d_\alpha^2/d_\beta^2 - 1\right)/\left(d_\alpha^2/d_\beta^2 + 1\right)\right]\left(1 - H_P\right)/d_P, \qquad [14]$$

$$B = 1 + 6.98\left(\frac{1 - H_P}{d_P}\right) \qquad [15]$$

and

$$X_0 = 0.964(1 - H_P)/d_P. \qquad [16]$$

These functional forms assume that d_P is dimensionless and given by $d_P = \frac{\hat{d}_P}{1\mu m}$, where $\hat{d}_P$ is the dimensional diameter. We maintain this convention throughout this section.

HS model with memory.

Simplifying assumptions. Before we explain how we extend the model from (18) to incorporate memory effects, we comment on its main simplifying assumptions. At present, our model does not include any information on local flow rate. Furthermore, the current model does not account for the angle defined by the planes containing the current and previous bifurcation in the network. These simplifying assumptions could easily be relaxed.

Rewriting of the model. In this section, we rewrite the HS model with memory effects ((2) from the main text) in terms of discharge haematocrit levels H and flow rates Q experienced by the vessels belonging to a given bifurcation ((3) from the main text). The definitions of $FQ_{E,f}$ and $FQ_{B,f}$ can be written as:

$$FQ_{E,f} = \frac{Q_f H_f}{Q_P H_P}, \qquad FQ_{B,f} = \frac{Q_f}{Q_P}.$$

Substituting these expressions into (2) from the main text gives:

$$\text{logit}\left(\frac{Q_f H_f}{Q_P H_P}\right) = A_f + B \ \text{logit}\left(\frac{Q_f/Q_P - X_{0,f}}{1 - X_{0,u} - X_{0,f}}\right).$$

Recalling that $\text{logit}(x) = \ln(x/(1-x))$, we have

$$\ln\left(\frac{Q_f H_f}{Q_P H_P - Q_f H_f}\right) = A_f + B \ \ln\left(\frac{Q_f - X_{0,f} Q_P}{Q_P - Q_f - X_{0,u} Q_P}\right).$$

Appealing to conservation of blood (overall)

$$Q_P = Q_f + Q_u$$

and RBCs (in particular)

$$Q_P H_P = Q_f H_f + Q_u H_u \qquad [17]$$

at diverging bifurcations, we arrive at

$$\ln\left(\frac{Q_f H_f}{Q_u H_u}\right) = A_f + B \ \ln\left(\frac{Q_f - X_{0,f} Q_P}{Q_u - X_{0,u} Q_P}\right).$$

This equation can also be written as

$$\ln\left(\frac{H_f}{H_u}\right) = A_f + B \ \ln\left(\frac{Q_f - X_{0,f} Q_P}{Q_u - X_{0,u} Q_P}\right) - \ln\left(\frac{Q_f}{Q_u}\right),$$

which yields

$$\frac{H_f}{H_u} = e^{A_f}\left(\frac{Q_f - X_{0,f} Q_P}{Q_u - X_{0,u} Q_P}\right)^B \frac{Q_u}{Q_f}.$$

Choice of parameter values and CFL recovery function. Here we introduce the functional forms for A_f, $X_{0,f}$ and $X_{0,u}$, using empirical data to justify our choices. Guided by the dependence of A on the network branching history described in (19) (see Figure 7 therein), we propose

$$A_f = A + A_{shift} f(l; d_P), \qquad [18]$$

where A is given in Eq. (14), the positive constant A_{shift} corresponds to the maximum CFL disruption effect, and the function $f(l; d_P)$ describes how the recovery of the CFL depends on the distance l to the previous bifurcation and the diameter d_P of the parent vessel*.
For parameter A, we only have access to the scattered data with respect to the regressor from (12, 19) (as opposed to the regressor from (18)), which reads

$$A = -6.96 \ln\left(\frac{d_\alpha}{d_\beta}\right)/d_P. \qquad [19]$$

Using the extreme values of A in these data (see Supplementary Figure S8c), we estimate $A_{shift} = 0.5$. Note that in branching networks with every pair of child vessels having equal radii, both (12) and (18) yield $A = 0$. Thus, for our networks, the choice of A does not affect A_f at all (see Eq. (18)).

*Consistency of the model requires that $A_u = A - A_{shift} f(l; d_P)$.

For simplicity, we model the CFL recovery using an exponential function

$$f(l; d_P) = e^{-\frac{l}{\omega d_P}} \ , \tag{20}$$

where ω controls the temporal dynamics of CFL recovery. From (20), we note that the CFL width is (approximately) 90% recovered at a distance $l_{90} = 10 d_P$ from the previous bifurcation (see also Figure 3g). Accordingly, we choose ω so that

$$0.1 = e^{-\frac{10}{\omega}} \implies \omega = \frac{10}{\ln(10)} \approx 4.$$

Guided by the dependence of $X_{0,f}$ on flow history described in (19), we propose

$$X_{0,f} = X_0 \left(1 - f(l; d_P)\right). \tag{21}$$

Assuming, as a first approximation, that $X_{0,f} + X_{0,u}$ is constant and independent of the distance to the previous bifurcation (see Figure 3g), we define

$$X_{0,u} = X_0 \left(1 + f(l; d_P)\right). \tag{22}$$

Validation of the HS model with memory. We validate the HS model with memory by comparing its predictions with results from the RBC simulations in the double-t geometry. We assume that all vessels have the same diameter ($d = 33$ μm), and that the flow rate splits evenly at both bifurcations. If we assume further that the CFL is fully established at the network inlet vessel, $H_{inlet} = 20\%$, then (1) from the main text supplies $H_1 = H_2 = H_{inlet} = 20\%$. We use conservation of RBCs Eq. (17) and the new HS model (Eq. (3) from the main text) to estimate haematocrit values in the unfavourable and favourable child branches after the second bifurcation (channels 3 and 4, respectively) for varying inter-bifurcation distances δ. The results are summarised in Supplementary Table S9. For $\delta = 4d$, the new HS model predicts haematocrits within 5% relative error of the values calculated from RBC simulations (Table 2). Given the uncertainty in determining discharge haematocrit in the RBC simulations and given that the new model neglects effects due to asymmetric streamline splitting (21), we conclude that our new model provides a good, leading-order approximation to the effects of CFL disruption on HS.

Finally, we compare the CFL evolution dynamics calculated from the RBC simulations (for $\theta = 0$ and $\theta = \pi$) with those predicted from the proposed evolution of $X_{0,f}$ and $X_{0,u}$ (Eq. (21) and Eq. (22)). In the absence of a known functional form relating the CFL width W and the minimum flow fraction X_0, we define

$$X_{0,f/u} = \frac{W_{f/u}}{d_P}. \tag{23}$$

Eq. (23) is based on the diagram in Supplementary Figure S8a and the assumptions of a cross-sectionally uniform velocity profile within a one-dimensional vessel cross-section. Combining Eq. (16), Eq. (23), Eq. (21) and Eq. (22), we conclude

$$W_{f/u} = d_P X_{0,f/u} = d_P X_0 \left(1 \mp f(l, d_P)\right) = 0.964 \times (1 - H_P) \left(1 \mp e^{-\frac{l}{4 \times d_P}}\right). \tag{24}$$

We remark that for a well-established CFL (i.e. $l \to \infty$), Eq. (24) predicts (noting that channel 1 serves as the parent vessel for the second bifurcation and estimating $H_P = 0.2$ from Supplementary Table S6) a CFL width of about 0.77 μm, whereas our RBC simulation predicts a value of approximately 1.8 μm (see Supplementary Figure S8b). We postulate that this discrepancy is caused by our oversimplification of the relationship between the CFL width and the minimum flow fraction (Eq. (23)). Nevertheless, we can adjust Eq. (24) so that it is consistent with the established CFL width of 1.8 μm by writing

$$W_{f/u} = 1.8 \times \left(1 \mp e^{-\frac{l}{4 \times d_P}}\right). \tag{25}$$

In this case the CFL evolution (for $\theta = 0$ and $\theta = \pi$) follows a trend similar to that observed in our RBC simulations (Supplementary Figure S8b). In particular, our assumption that $l_{90} = 10 d_P$ is in good agreement with our simulation results (see dashed line in Supplementary Figure S8b).

Explanation of higher mean oxygen values for small λ. We observed that CFL disruption effects increase the mean oxygen concentration in the chosen network (Supplementary Figure S6). Here, we provide an explanation of this phenomenon.

We define

$$\Delta_\alpha H = H_\alpha - H_P, \quad \Delta_\beta H = H_\beta - H_P, \tag{26}$$

where P is the parent branch and α and β are the child branches of any diverging bifurcation. Conservation of blood and RBCs at this bifurcation then yields

$$Q_\alpha + Q_\beta = Q_P \tag{27}$$

$$Q_\alpha \left(H_P + \Delta_\alpha H\right) + Q_\beta \left(H_P + \Delta_\beta H\right) = Q_P H_P. \tag{28}$$

Combining Eq. (28) and Eq. (27) supplies

$$\frac{Q_\alpha}{Q_\beta} = -\frac{\Delta_\beta H}{\Delta_\alpha H}. \tag{29}$$

We deduce that, at diverging bifurcations, the haematocrit level in the child branch with higher flow rate deviates less (in absolute value) from the haematocrit in the parent vessel than the branch with lower flow rate.

We note further that all paths connecting the inlet and outlet vessels in the direction of blood flow in a given network are topologically and geometrically equivalent. Therefore, heterogeneity in haematocrit splitting arises solely from CFL disruption effects. If haematocrit is elevated in one of the child branches, its impedance will increase, and, as a result, it will receive a lower flow rate.

Combining these two effects, we see that, in the chosen networks, haemoconcentration in any child branch is more significant than haemodilution in its sibling. As a consequence, and given that the strength of the oxygen source term in Eq. (13) is a linear function of H, we observe higher mean oxygen levels when the effects of CFL disruption are taken into account (especially for small λ). Future work will investigate this effect by making source term a function of RBC mass flux (*i.e.* QH) or relaxing the assumption that the RBCs have infinite oxygen carrying capacity.

Table S1. Average vessel lengths and diameters reported in a variety of tissues under physiological conditions.

Animal (tissue)	Vessel type	$\bar{L}(\mu\text{m})$	$\bar{d}(\mu\text{m})$	$\lambda = \bar{L}/\bar{d}$	Reference
Wistar Kyoto Rat (mesentery)	Arteriole	337.0	13.2	25.5	(22, 23)
	Capillary	424.0	8.7	48.7	(22, 23)
	Venule	334.0	20.6	16.2	(22, 23)
Myotis Bat (wing)	Arteriole	206.0	7.0	29.4	(24)
	Capillary	74.0	3.7	20.0	(25)
	Venule	200.0	21.0	9.5	(25)
Cat (sartorius muscle)	Arteriole	96.0	7.4	13.0	(26)
	Venule	68.0	6.8	10.0	(27)
Golden Hamster (retractor muscle)	Arteriole	101.2	5.7	17.8	(28)
	Venule	57.7	3.6	16.0	(28)
Human	Capillary	350.0	5.0	70.0	(29)

Table S2. Previous studies reporting morphological or haemodynamic changes following vascular normalisation therapy.

Ref.	Model	Treatment	Changes reported	λ ratio implications	Efficacy
(30)	MDA-MB-361HK tumour model	Herceptin	Reduced vessel diameter, unchanged length	λ increase	Tumour growth slowed and increased survival
(31)	Shionogi tumour model	Androgen withdrawal	Reduced vessel diameter	Possible λ increase	Tumour regression followed by relapse
(32)	Glioblastoma tumour model	Cediranib	Transient reduction in vessel diameter and haemoconcentration	Possible λ increase	Increased survival
(33)	Rip-Tag2 tumour model	Anti-α6 integrin antibody	Reduced vessel diameter	Possible λ increase	Impaired tumour angiogenesis
(34)	Murine mammary carcinoma MCaIV	DC101	Relatively higher reduction in diameter than in length	λ increase	Increased drug penetration via functional normalisation
(35)	LS174T tumour model	Anti-VGF antibody (A4.6.1)	Reduced vessel diameter, unchanged length	λ increase	Vascular regression and decreased permeability

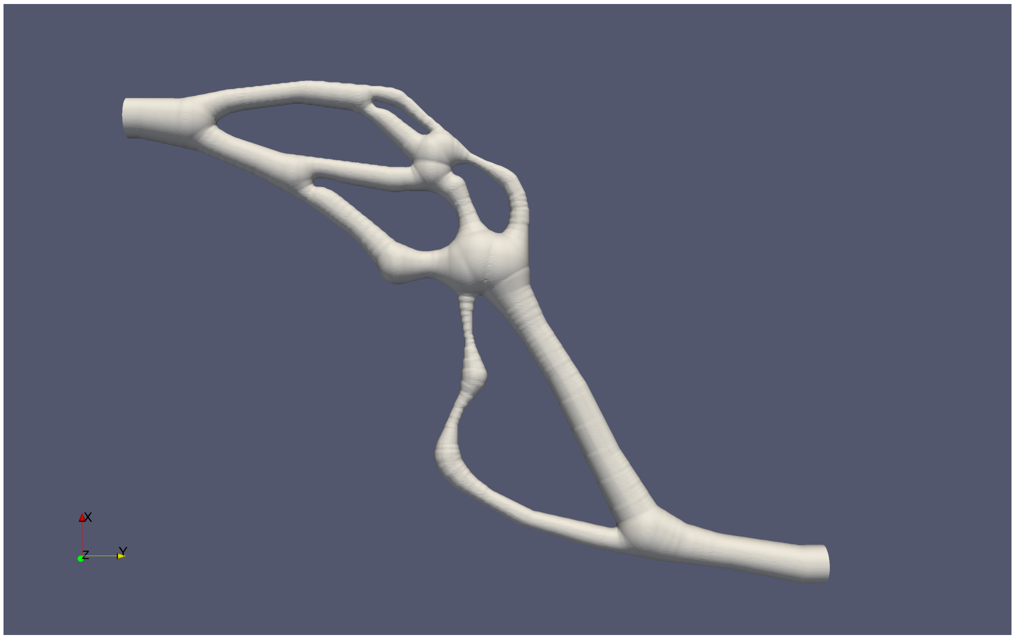

Fig. S1. Realistic capillary network reconstructed from MC38 tumour vascular network.

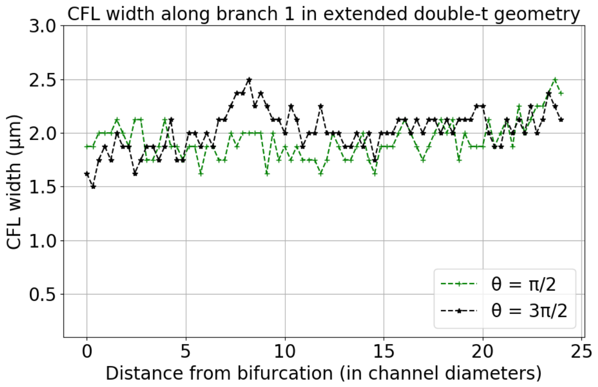

Fig. S2. CFL channel 1 double-t geometry perpendicular to bifurcation planes.

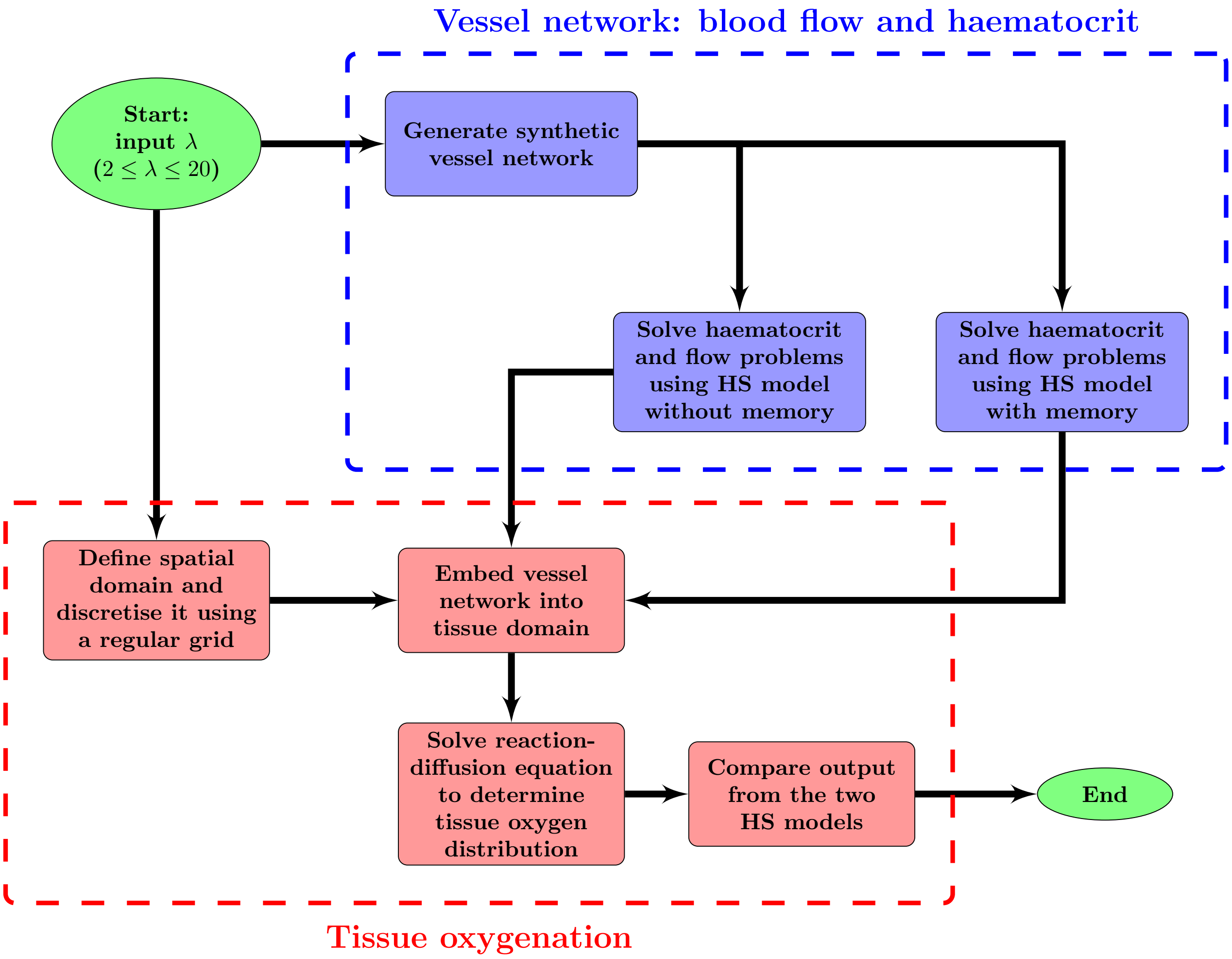

Fig. S3. Flow chart summarising the main components of our hybrid model for tissue oxygen perfusion, as implemented within Microvessel Chaste (see (15)).

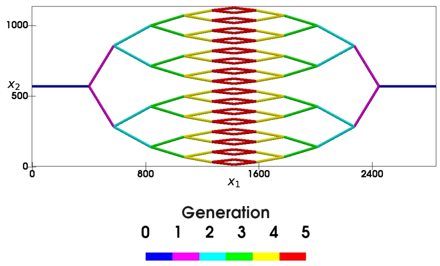

(a) A typical forking vessel network

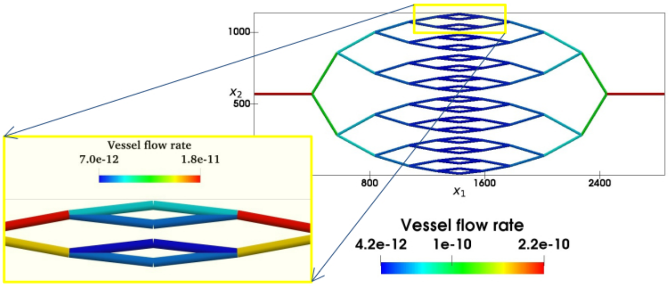

(b) Vessel flow rates (in m^3/s)

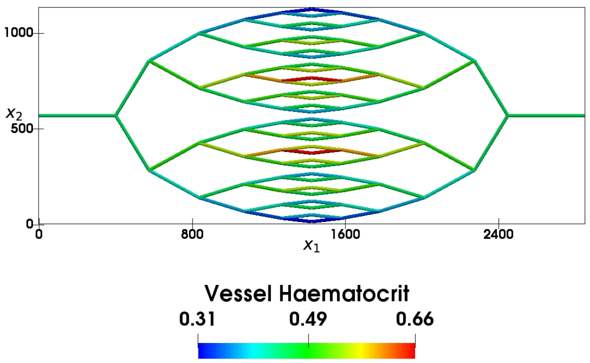

(c) Distribution of haematocrit across vessel network (model with memory effects)

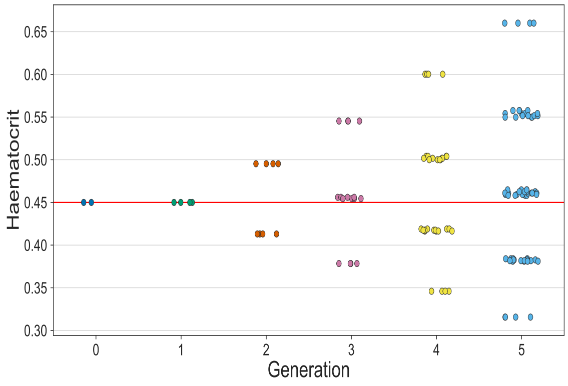

(d) Propagation of memory effects

Fig. S4. (a) A typical symmetric forking network with 6 generations of vessels. (b) Flow rates almost halve between consecutive vessel generations. However, small differences in flow rates between child vessels arise due to non-uniform haematocrit splitting (HS), as can be observed in the inset (note that the range of the colour bar has been adjusted to represent only the selected vessels). (c) Differences in the predicted haematocrit levels of child vessels (within a single vessel generation) become more pronounced as the generation number increases. (d) For the new HS model, the haematocrit distribution becomes more disperse as the number of bifurcations included in the network increases (the red horizontal line represents the predicted haematocrit when memory effects are neglected and haematocrit is distributed uniformly across the network). Each circle corresponds to a single vessel and different colours correspond to different vessel generations.

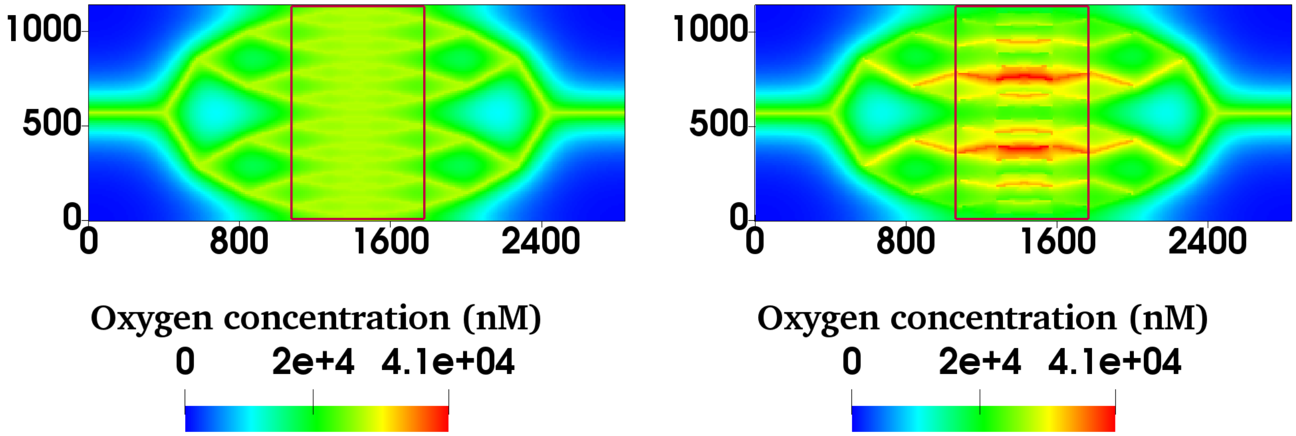

(a) $\lambda = 4$, model without memory effects

(b) $\lambda = 4$, model with memory effects

Fig. S5. For $\lambda = 4.0$, the model with memory effects yields more pronounced oxygen heterogeneity (*i.e.* more dispersed oxygen distribution) in the region of interest bounded by red rectangles in (a) and (b) (note that the spatial scales are in microns).

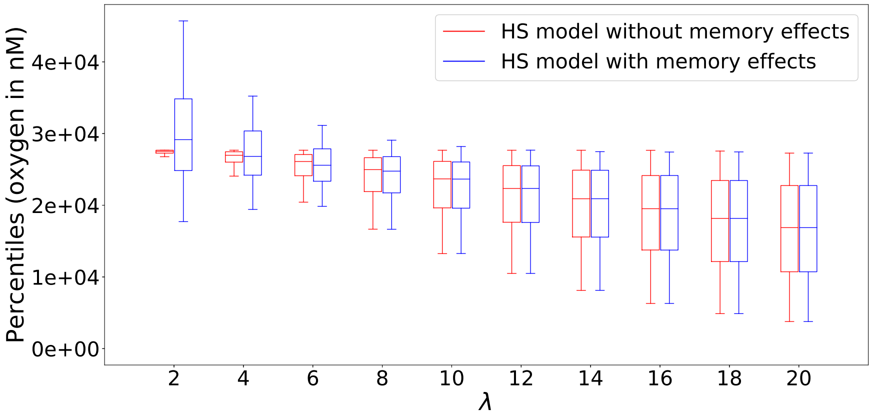

(a) Boxplots showing tissue oxygen concentration distribution as a function of λ

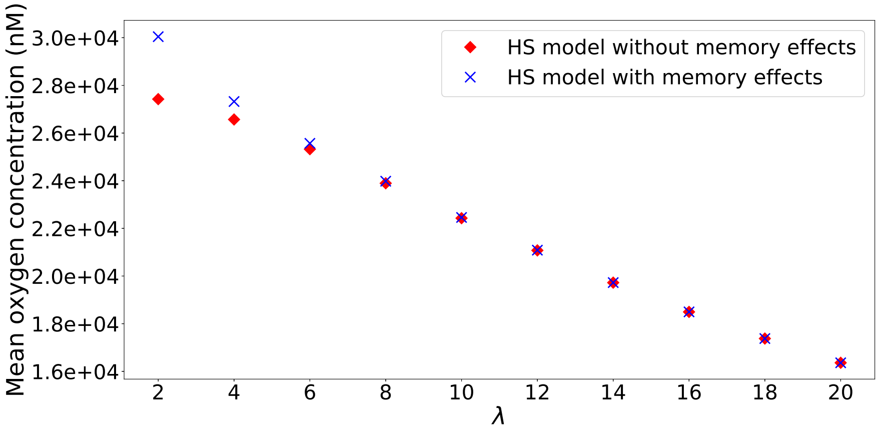

(b) Mean oxygen concentration as a function of λ

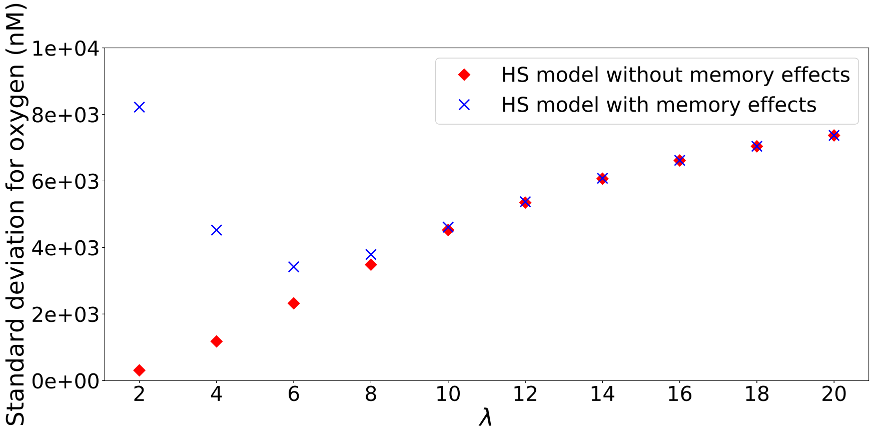

(c) Standard deviation (oxygen) as a function of λ

Fig. S6. Summary statistics illustrating how for a vessel network with 6 generations its λ value and the HS model affect tissue oxygenation. (a) Boxplots showing how the tissue oxygen distribution changes as λ varies for the two different HS rules. (b) Mean oxygen concentration increases as λ decreases (and the vessel density increases). (c) Standard deviation in the tissue oxygen concentration increases with λ when memory effects are neglected ((1) from the main text). When memory effects are considered ((2) from the main text), the standard deviation increases for small λ values. The mean and standard deviation for the two models converge for large λ values. Model parameter values as per Supplementary Table S8.

Table S3. λ **values measured in MC38 tumours following DC101 treatment over time.**

Day	Mouse 1	Mouse 2	Mouse 3	Mouse 4	Mouse 5
0	4.532	4.065	4.141	4.122	4.054
1	5.301	4.098	4.336	4.432	3.878
2	6.222	4.429	4.396	5	4.756
3	5.382	4.465	3.89	5.353	5.068
4	4.395	4.418	3.273	6.342	4.237

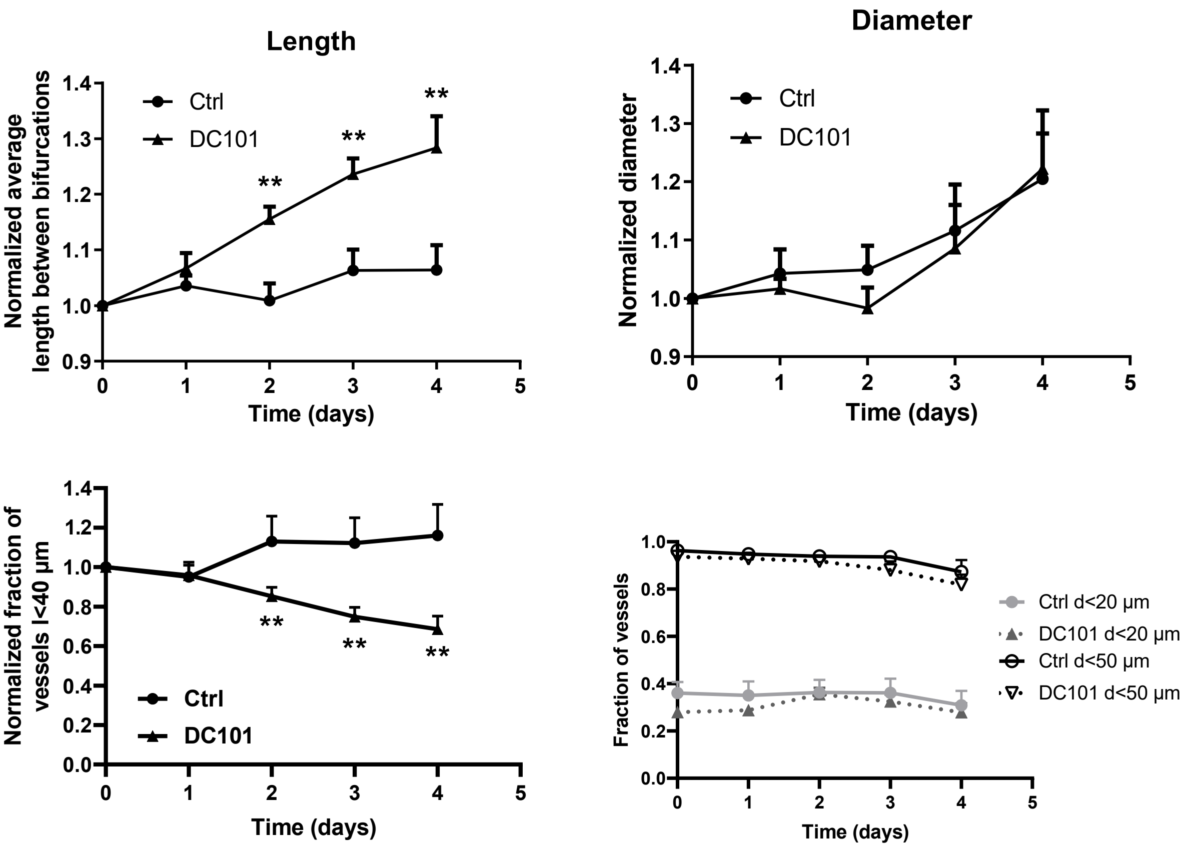

Fig. S7. Vascular phenotypes in MC38 tumours over time following DC101 treatment compared with control (n=5). * $p < 0.05$, ** $p < 0.01$.

Table S4. Timecourse of Pearson's r-values calculated for different mice at different days of measurement implanted with the MC38 cell line. Day 0 corresponds to the day of the first measurement, when the tumour reached a specified size (4mm in diameter; see the main text). The corresponding values of $\bar{L}$, $\bar{d}$ and λ are reported in the main text (see Table 1). The missing datum for tumour 3 on Day 3 is due to the laser on the microscope failing during imaging.

Day	1	2	3	4	5	6
0	0.05	-0.06	-0.07	0.10	-0.13	0.00
1	0.03	-0.05	-0.07	-0.07	-0.13	-0.00
2	-0.08	-0.07	-0.06	-0.17	-0.19	0.02
3	-0.09	-0.11	-	-0.14	-0.13	-0.02
4	-0.14	-0.09	-0.08	-0.17	-0.12	-0.07
5	-	-0.04	-	-	-0.07	-0.11
6	-	-0.08	-	-	-	-

Table S5. Timecourse of Pearson's r-values for mice implanted with the B16F10 and LLC cell lines. Day 0 corresponds to the day of the first measurement, when the tumour reached a specified size (4mm in diameter; see the main text). The corresponding values of $\bar{L}$, $\bar{d}$ and λ are reported in the main text (see Table 1).

	B16F10			LLC
Day	1	2	3	
0	-0.08	-0.08	-0.06	0.03
1	-0.05	-0.06	-0.09	0.02
2	-0.05	-0.12	-0.06	-
3	-0.03	-0.11	-0.06	-
4	-	-	-0.06	-
5	-	-	-0.05	-

Table S6. Parameters in RBC simulations in synthetic capillary networks

Parameter	Description	Value	Reference
d	Cylindrical channel diameter	33 μm	Current study
L'	Inlet/outlet channel length	$25d$	(20)
δ	Distance between branching points	$4d, 25d$	(20), current study
$\bar{v}_{inlet}$	Inlet mean velocity	600 μm/s	(36)
H_{inlet}	Inlet discharge haematocrit	20%	(36)

Table S7. Parameters used in RBC simulation algorithm. The number of faces is chosen in such a way that the average edge length of a triangular element matches the grid spacing of the fluid lattice. The value of the capillary number is representative of typical flows in the microcirculation. The adopted value of the Föppl-von Kármán number matches the intrinsic property of healthy RBC membranes. The remaining moduli are chosen in such a way that the local area, total surface area and volume of the RBCs are constrained within a few percent while simulations remain numerically stable.

Parameter	Description	Value	Unit	Comment
N_f	Number of faces	500	-	See mesh convergence analysis in (6)
r_{RBC}	RBC diameter	8	μm	6.2–8.2 μm physiological range
η	Plasma viscosity	1	mPa s	Approximated by water
Ca	Capillary number	0.1	-	$Ca = \frac{\eta \dot{\gamma} r_{RBC}}{\kappa_s}$, $\dot{\gamma} = \frac{\bar{v}_{inlet}}{d}$ (see Suppl. Table S6, (20))
Γ	Föppl-von Kármán no.	400	-	$\Gamma = \frac{\kappa_s {r_{RBC}}^2}{\kappa_B}$
κ_α	Dilation modulus	0.5	-	Strong volume and area conservation
κ_A	Surface area modulus	1	-	Strong volume and area conservation
κ_V	Volume modulus	1	-	Strong volume and area conservation

Table S8. Parameters used to simulate tissue oxygen.

Parameter	Description	Value	Unit	Reference
D	Diffusivity	0.00145	cm^2 min^{-1}	(37)
κ	Consumption rate	13.0	min^{-1}	(37)
γ	Vessel permeability	6.0	cm min^{-1}	(37)
c_{stp}	Ideal gas concentration	$\frac{1}{0.0224}$	mol m^{-3}	(38)
p_{ref}	Reference partial pressure	20	mmHg	(37)
α_{eff}	Volumetric solubility	3.1×10^{-5}	mmHg^{-1}	(17)
H_{inlet}	Inlet haematocrit	0.45	-	(39)
d_{inlet}	Diameter of inlet vessel	100	μm	Estimated from (13)
A_{shift}	Maximum CFL disruption effect	0.5	-	Estimated here
ω	Temporal dynamics of CFL recovery	4	-	Estimated here
μ_p	Plasma viscosity	10^{-3}	Poiseuille	Similar to (37)
p_{in}	Inlet pressure	3.32×10^3	Pa	Similar to (37)
p_{out}	Outlet pressure	2.09×10^3	Pa	Similar to (37)

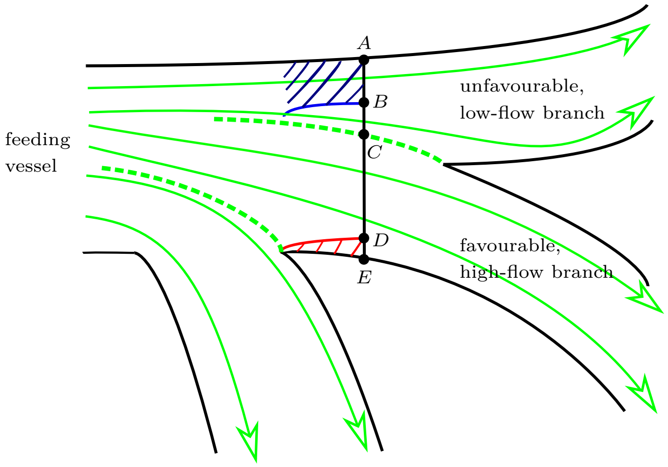

(a) Flow and haematocrit separation

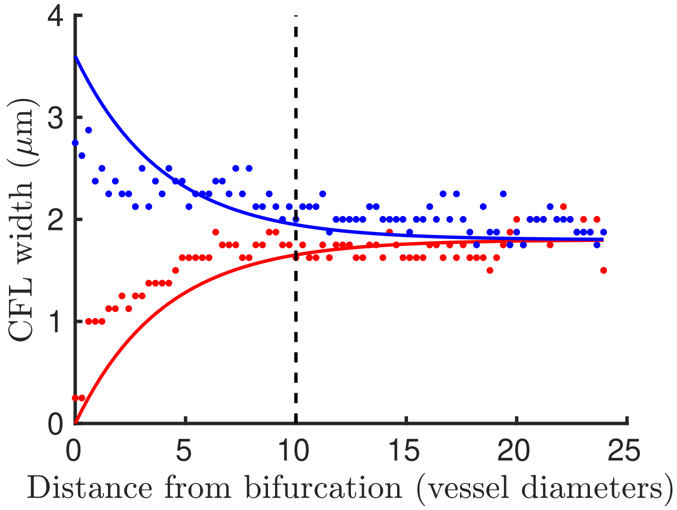

(b) CFL recovery

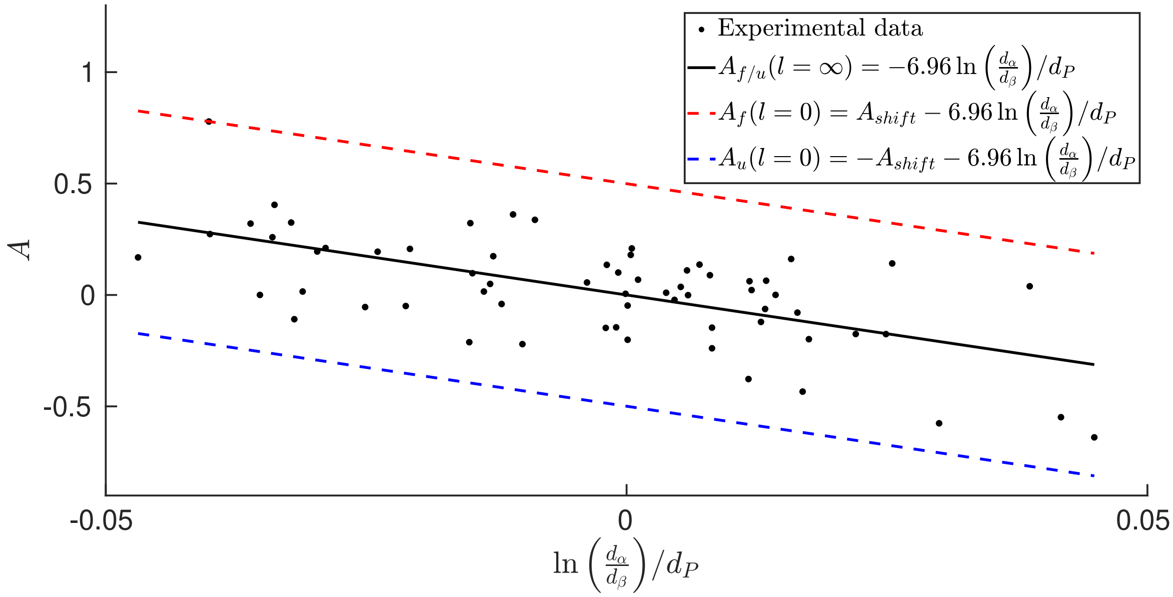

(c) Dispersion of data for A from (19)

Fig. S8. (a) A schematic diagram presenting the geometric intuition behind (blood) flow and haematocrit separation illustrate why two distinct minimum-flow fractions are needed to characterise the favourable and the unfavourable 2 Synthetic vascular networksbranches: $\|AB\| = X_{0,u}$, $\|DE\| = X_{0,f}$, $\|AC\| = FQ_{B,u}$ and $\|CE\| = FQ_{B,f}$. Blood flow separation at the two consecutive bifurcations is shown in dotted green, streamlines are sketched with yellow curved arrows, and the CFL recovery on the favourable (unfavourable) side of the parent vessel after the first bifurcation is sketched in red (blue). Whenever $FQ_{B,f} < X_{0,f}$ ($FQ_{B,u} < X_{0,u}$), the favourable (unfavourable) branch only draws blood from the CFL and it thus receives pure plasma. (b) Model of CFL recovery as described by Eq. (25) shows similar trends to and is in satisfactory agreement with the CFL width data from RBC simulations in Figure 3g (given the simplifying assumptions). The established CFL width of 1.8 μm chosen by inspection for this particular dataset. (c) Dispersion of values for A (reproduced using Figure 6 from (19)) is used with the regression from (12) to estimate the value of $A_{shift} \approx 0.5$ in Eq. (18), based on deviation from the regression. We assume the CFL disruption to be the primarily cause of this deviation, and thus its maximum (absolute) value should correspond to $l = 0$ in Eq. (20) (i.e. $f = 1$ in Eq. (18)).

Table S9. Haematocrits predicted by the model with CFL memory effects

Distance	H_{inlet}	H_u	H_f
$\delta = 4d$	20.0	17.7	22.3
$\delta = 11d$	20.0	19.6	20.4
$\delta = 18d$	20.0	19.9	20.1
$\delta = 25d$	20.0	20.0	20.0

References

1. Z Guo, C Zheng, B Shi, Discrete lattice effects on the forcing term in the lattice Boltzmann method. *Phys. Rev. E - Stat. Nonlinear, Soft Matter Phys.* **65**, 1–6 (2002).
2. M Bouzidi, M Firdaouss, P Lallemand, Momentum transfer of a Boltzmann-lattice fluid with boundaries. *Phys. Fluids* **13**, 3452 (2001).
3. AJC Ladd, Numerical simulations of particulate suspensions via a discretized Boltzmann equation. Part 1. Theoretical foundation. *J. Fluid Mech.* **271**, 285 (1994).
4. CK Aidun, JR Clausen, Lattice-Boltzmann Method for Complex Flows. *Annu. Rev. Fluid Mech.* **42**, 439–472 (2010).
5. T Krüger, et al., *The Lattice Boltzmann Method: Principles and Practice.* p. 694 (2017).
6. T Krüger, *Computer simulation study of collective phenomena in dense suspensions of red blood cells under shear.* (Springer Spektrum), p. 179 (2012).
7. R Skalak, PI Branemark, Deformation of Red Blood Cells in Capillaries. *Science* **164**, 717–719 (1969).
8. T Krüger, F Varnik, D Raabe, Efficient and accurate simulations of deformable particles immersed in a fluid using a combined immersed boundary lattice Boltzmann finite element method. *Comput. Math. with Appl.* **61**, 3485–3505 (2011).
9. CS Peskin, The immersed boundary method in *Acta Numerica*, ed. A Iserles. (Cambridge University Press, Cambridge) Vol. 11, pp. 479–518 (2002).
10. G Gompper, M Schick, *Soft Matter: Lipid bilayers and red blood cells* eds. G Gompper, M Schick. (Wiley), p. 254 (2008).
11. CD Murray, The Physiological Principle of Minimum Work: I. The Vascular System and the Cost of Blood Volume. *Proc. Natl. Acad. Sci.* **12**, 207–214 (1926).
12. AR Pries, TW Secomb, P Gaehtgens, JF Gross, Blood flow in microvascular networks. Experiments and simulation. *Circ. Res.* **67**, 826–34 (1990).
13. TW Secomb, Hemodynamics. *Compr. Physiol.* **6**, 975–1003 (2016).
14. AR Pries, et al., Resistance to blood flow in microvessels in vivo. *Circ. Res.* **75**, 904–915 (1994).
15. JA Grogan, et al., Microvessel Chaste: An Open Library for Spatial Modeling of Vascularized Tissues. *Biophys. J.* **112**, 1767–1772 (2017).
16. T Alarcón, HM Byrne, PK Maini, A cellular automaton model for tumour growth in inhomogeneous environment. *J. Theor. Biol.* **225**, 257–274 (2003).
17. TW Secomb, R Hsu, EYH Park, MW Dewhirst, Green ' s Function Methods for Analysis of Oxygen Delivery to Tissue by Microvascular Networks. *Annals biomedical engineering* **32**, 1519–1529 (2004).
18. AR Pries, TW Secomb, Microvascular blood viscosity in vivo and the endothelial surface layer. *Am. J. Physiol. Circ. Physiol.* **289**, H2657–H2664 (2005).
19. A Pries, K Ley, M Claassen, P Gaehtgens, Red cell distribution at microvascular bifurcations. *Microvasc. Res.* **38**, 81–101 (1989).
20. D Katanov, G Gompper, DA Fedosov, Microvascular blood flow resistance: Role of red blood cell migration and dispersion. *Microvasc. Res.* **99**, 57–66 (2015).
21. Z Wang, Y Sui, AV Salsac, D Barthès-Biesel, W Wang, Path selection of a spherical capsule in a microfluidic branched channel: Towards the design of an enrichment device. *J. Fluid Mech.* **849**, 136–162 (2018).
22. BR Duling, IH Sarelius, WF Jackson, A comparison of microvascular estimates of capillary blood flow with direct measurements of total striated muscle flow. *Int. journal microcirculation, clinical experimental* **1**, 409–24 (1982).
23. AS Popel, A Model of Pressure and Flow Distribution in Branching Networks. *J. Appl. Mech.* **47**, 247 (1980).
24. MP Wiedeman, Lengths and Diameters of Peripheral Arterial Vessels in the Living Animal. *Circ. Res.* **10**, 686–690 (1962).
25. MP Wiedeman, Dimensions of Blood Vessels from Distributing Artery to Collecting Vein. *Circ. Res.* **12**, 375–378 (1963).
26. A Koller, B Dawant, A Liu, AS Popel, PC Johnson, Quantitative analysis of arteriolar network architecture in cat sartorius muscle. *Am. J. Physiol. Circ. Physiol.* **253**, H154–H164 (1987).
27. SD House, PC Johnson, Diameter and blood flow of skeletal muscle venules during local flow regulation. *Am. J. Physiol. Circ. Physiol.* **250**, H828–H837 (1986).
28. M Ellsworth, A Liu, B Dawant, A Popel, R Pittman, Analysis of vascular pattern and dimensions in arteriolar networks of the retractor muscle in young hamsters. *Microvasc. Res.* **34**, 168–183 (1987).
29. AR Pries, TW Secomb, Rheology of the microcirculation. *Clin. Hemorheol. Microcirc.* **29**, 143–8 (2003).
30. Y Izumi, L Xu, E di Tomaso, D Fukumura, RK Jain, Herceptin acts as an anti-angiogenic cocktail. *Nature* **416**, 279–280 (2002).
31. RK Jain, et al., Endothelial cell death, angiogenesis, and microvascular function after castration in an androgen-dependent tumor: role of vascular endothelial growth factor. *Proc. Natl. Acad. Sci. United States Am.* **95**, 10820–5 (1998).
32. WS Kamoun, et al., Edema control by cediranib, a vascular endothelial growth factor receptor - Targeted kinase inhibitor, prolongs survival despite persistent brain tumor growth in mice. *J. Clin. Oncol.* **27**, 2542–2552 (2009).
33. L Primo, et al., Increased expression of α6 integrin in endothelial cells unveils a proangiogenic role for basement membrane. *Cancer Res.* **70**, 5759–5769 (2010).
34. RT Tong, et al., Vascular normalization by vascular endothelial growth factor receptor 2 blockade induces a pressure gradient across the vasculature and improves drug penetration in tumors. *Cancer Res.* **64**, 3731–3736 (2004).
35. F Yuan, et al., Time-dependent vascular regression and permeability changes in established human tumor xenografts induced by an anti-vascular endothelial growth factor/vascular permeability factor antibody. *Proc. Natl. Acad. Sci.* **93**,

14765–14770 (1996).

36. WS Kamoun, et al., Simultaneous measurement of RBC velocity, flux, hematocrit and shear rate in vascular networks. *Nat. Methods* **7**, 655–660 (2010).
37. MR Owen, et al., Mathematical modeling predicts synergistic antitumor effects of combining a macrophage-based, hypoxia-targeted gene therapy with chemotherapy. *Cancer Res.* **71**, 2826–2837 (2011).
38. HNS Michael J. Moran, *Fundamentals of Engineering Thermodynamics.* p. 1042 (2006).
39. AR Pries, TW Secomb, P Gaehtgens, Biophysical aspects of blood flow in the microvasculature. *Cardiovasc. research* **32**, 654–67 (1996).
